# New inhibitors of cathepsin V impair tumor cell proliferation and elastin degradation and increase immune cell cytotoxicity

**DOI:** 10.1016/j.csbj.2022.08.046

**Published:** 2022-08-28

**Authors:** Ana Mitrović, Emanuela Senjor, Marko Jukić, Lara Bolčina, Mateja Prunk, Matic Proj, Milica Perišić Nanut, Stanislav Gobec, Janko Kos

**Affiliations:** a Department of Biotechnology, Jožef Stefan Institute, Jamova 39, 1000 Ljubljana, Slovenia; b Faculty of Pharmacy, University of Ljubljana, Aškerčeva cesta 7, 1000 Ljubljana, Slovenia

**Keywords:** 7-AAD, 7-aminoactinomycin D, ATCC, American Type Culture Collection, AUC, area under curve, CFSE, carboxyfluorescein succinimidyl ester, CTLs, cytotoxic T lymphocytes, DMEM, Dulbecco’s modified Eagle’s medium, E:T, effector-to-target ratio, E2F1, E2 promoter-binding factor 1, FBS, fetal bovine serum, LHVS, morpholinurea-leucine-homophenylalanine-vinylsulfone-phenyl, MDCK, Madin−Darby Canine Kidney cells, MTS, 3-(4,5-dimethylthiazol-2-yl)-5-(3-carboxymetoxyphenyl)-2-(4-sulfophenyl)–2H-tetrazolium, NK, natural killer, PMA, phorbol 12-myristate 13-acetate, RMSD, Root Mean Square Deviation, Cathepsin V, Small-Molecule Inhibitors, Antitumor therapy, Cancer, Cystatin F

## Abstract

Cathepsin V is a human lysosomal cysteine peptidase with specific functions during pathological processes and is as such a promising therapeutic target. Peptidase inhibitors represent powerful pharmacological tools for regulating excessive proteolytic activity in various diseases. Cathepsin V is highly related to cathepsin L but differs in tissue distribution, binding site morphology, substrate specificity, and function. To validate its therapeutic potential and extend the number of potent and selective cathepsin V inhibitors, we used virtual high-throughput screening of commercially available compound libraries followed by an evaluation of kinetic properties to identify novel potent and selective cathepsin V inhibitors. We identified the ureido methylpiperidine carboxylate derivative, compound **7**, as a reversible, selective, and potent inhibitor of cathepsin V. It also exhibited the most preferable characteristics for further evaluation with *in vitro* functional assays that simulate the processes in which cathepsin V is known to play an important role. Compound **7** exerted significant effects on cell proliferation, elastin degradation, and immune cell cytotoxicity. The latter was increased because compound **7** impaired conversion of immunosuppressive factor cystatin F to its active monomeric form. Taken together, our results present novel potent inhibitors of cathepsin V and provide new hit compounds for detailed development and optimization. Further, we demonstrate that cathepsin V is a potential target for new approaches to cancer therapy.

## Highlights

• Identification of novel potent inhibitors of lysosomal cysteine peptidase cathepsin V.
• New inhibitors of cathepsin V demonstrated antitumor activity.
• They impair tumor cell proliferation and elastase degradation and increase immune cell cytotoxicity.
• Cathepsin V inhibitor impaired conversion of immunosuppressive factor cystatin F to its active monomeric form.

## Graphical abstract

**Figure:**
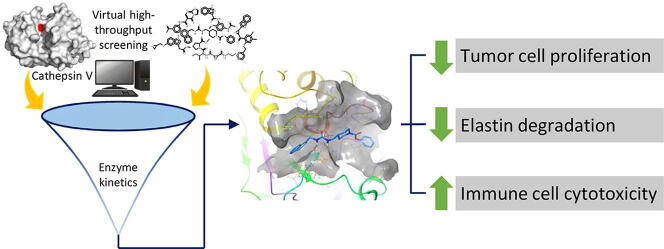

## 1 Introduction

Cathepsin V (also known as cathepsin L2) is a human lysosomal cysteine peptidase that belongs to clan CA of the papain family (C1) [1]. As are most cysteine cathepsins, cathepsin V is an endopeptidase. It is highly related to cathepsin L, sharing 80 % primary sequence identity [2], [3], [4]. No mouse orthologue has been found for cathepsin V; however, great similarities were found between human cathepsin V and mouse cathepsin L, suggesting that human cathepsin V is functionally equivalent to mouse cathepsin L [3], [4]. Moreover, the genes encoding both cathepsins V and L are located at the same human chromosomal region, 9q22.2 [4]. Despite high homology between the two cathepsins, they differ in their tissue distribution, binding site morphology, substrate specificity, and function [2], [3], [4], [5], [6]. In contrast to ubiquitously expressed cathepsin L, cathepsin V is localized mainly within the thymus, corneal epithelium, and testis under normal physiological conditions [2], [3], [4].

Structurally, cathepsin V is similar to other cysteine peptidases of the papain family; however, it differs markedly in its 10-residue surface loop, a structure that typically varies among the cathepsins [5]. Cathepsin V is *N*-glycosylated at amino acid residues Asn221 and Asn292, which seems to be important for its localization and function [7]. Interestingly, despite extensive sequence identity and structural similarities, cathepsins V and L remarkably differ in their surface electrostatic potentials [4]. Cathepsins V and L also differ in their binding site morphologies [4], [5] and substrate specificities [6]. Cathepsin V has potent elastolytic activity [8] and is important for invariant chain processing during MHC-II antigen presentation within the thymus [9]. In addition, cathepsin V is involved in the processing and activation of human pro-cathepsin C [10] and in the monomerization and activation of cystatin F in cytotoxic granules of natural killer (NK) cells and cytotoxic T lymphocytes (CTLs) [11]. Moreover, cathepsin V participates in the production of neuropeptides in the brain [12], [13].

Elevated cathepsin V levels have been associated with various pathological processes, including myasthenia gravis [9], atherosclerosis [8], vascular inflammation [14], type 1 diabetes [15], mucopolysaccharidoses [16], neurological diseases [12], and cancer [3]. In cancer, high cathepsin V expression was first detected in colorectal and breast carcinomas [3], and later also in ovarian, endometrial, renal, squamous cell, thymic epithelial [17], hepatocellular, and thyroid carcinomas [18], [19]. Higher cathepsin V levels were associated with increasing tumor stage [20], [21], distant metastases [22], [23], [24], and lower patient survival [24]. Taken together, these results indicate that cathepsin V could be used as a diagnostic and prognostic marker for cancer [3], [20], [21], [22], [23], [24].

Cathepsin V overexpression in various carcinomas correlates with cell hyperproliferation [3], [18], [20], [24]. The function of cathepsin V to trigger hyperproliferation could be linked with its localization within the nucleus [18]. In breast cancer, cathepsin V suppresses the expression of GATA3, a member of the zinc finger transcription factor family, by facilitating its turnover via the proteasome [24]. Furthermore, cathepsin V expression is induced by direct binding of E2 promoter-binding factor 1 (E2F1) to its promoter, which is required for E2F1-induced apoptosis [25]. E2F1 activates the transcription of genes required for DNA replication and plays a fundamental role in cell cycle regulation and cell proliferation in various tumor types, indicating its oncogenic function [18], [25]. In thyroid cancer, E2F1 overexpression can lead to cathepsin V upregulation [18]. In colorectal cancer cells, cathepsin V function can be regulated by tumor-suppressive tazarotene-induced gene 1, which decreases the stability of cathepsin V and promotes its degradation, resulting in lower cell invasion and migration [26]. Cathepsin V also increases the levels of activated urokinase-type plasminogen activator and alters the expression of proteins associated with epithelial-mesenchymal transition [26]. In addition, cathepsin V, induced by l-homocysteine, promotes the expression of inflammatory cytokines [14]. Cathepsin V also participates in pyroptosis of endothelial cells during vascular endothelial inflammation by mediating caspase-1 activation induced by high-mobility group box-1 protein [27].

Cathepsin V impairs the antitumor immune response by converting cystatin F, a cysteine peptidase inhibitor, from its inactive dimer to its active monomer by proteolytic cleavage of the 15-amino-acid *N*-terminal peptide [11], [28]. Active cystatin F is a potent inhibitor of cathepsins C and H, the main granzyme convertases in cytotoxic immune cells [29], [30]. Its elevated levels in NK cells and CTLs induce anergy, a state in which cells lose their cytotoxicity [31], [32]. Cystatin F is secreted into the tumor microenvironment by tumor and other immune cells. It represents a potent immunosuppressive factor that can be internalized into NK cells and CTLs, inactivating their cytotoxicity [28], [33] by modulating their endo/lysosomal cysteine cathepsins.

Due to its role in cancer progression, cathepsin V is considered a target for antitumor treatment [24]. However, only few cathepsin-V-selective inhibitors have been identified so far. Soon after its identification, recombinant cathepsin V was shown to be strongly inhibited by general cysteine peptidase inhibitors, such as E-64, cystatins, peptidyl vinyl sulfones, and iodoacetic acid [4]. Later, some natural products were described as cathepsin V inhibitors, e.g., macrocypins [34], natural flavones and their synthetic derivatives [35], acridione alkaloids isolated from *Swinglea glutinosa* [36], and dimeric chalcone derivatives [37]. Based on the structure of acridone alkaloid inhibitors, new synthetic *N*-arylanthranilic acid, acridone, and 4-quinolone inhibitors were prepared as cathepsin V and L inhibitors [38]. These compounds showed strong inhibition of cathepsins V and L, and only *N*-arylanthranilic acid derivatives showed a moderate selectivity for cathepsin V [38].

To expand the current array of potent and selective cathepsin V inhibitors, we screened a library of 13.7 million compounds for their selective binding to cathepsin V using virtual screening, molecular docking, and subsequent biochemical evaluation. We then further evaluated the hit compounds that best inhibited cathepsin V in biological processes. For this, we developed cell-based *in vitro* functional assays to simulate processes in which cathepsin V is known to play an important role: elastin degradation, tumor cell proliferation, and immune cell cytotoxicity.

## 2 Materials and methods

### 2.1 Preparation of chemical library for virtual screening

**Fig. 1:**
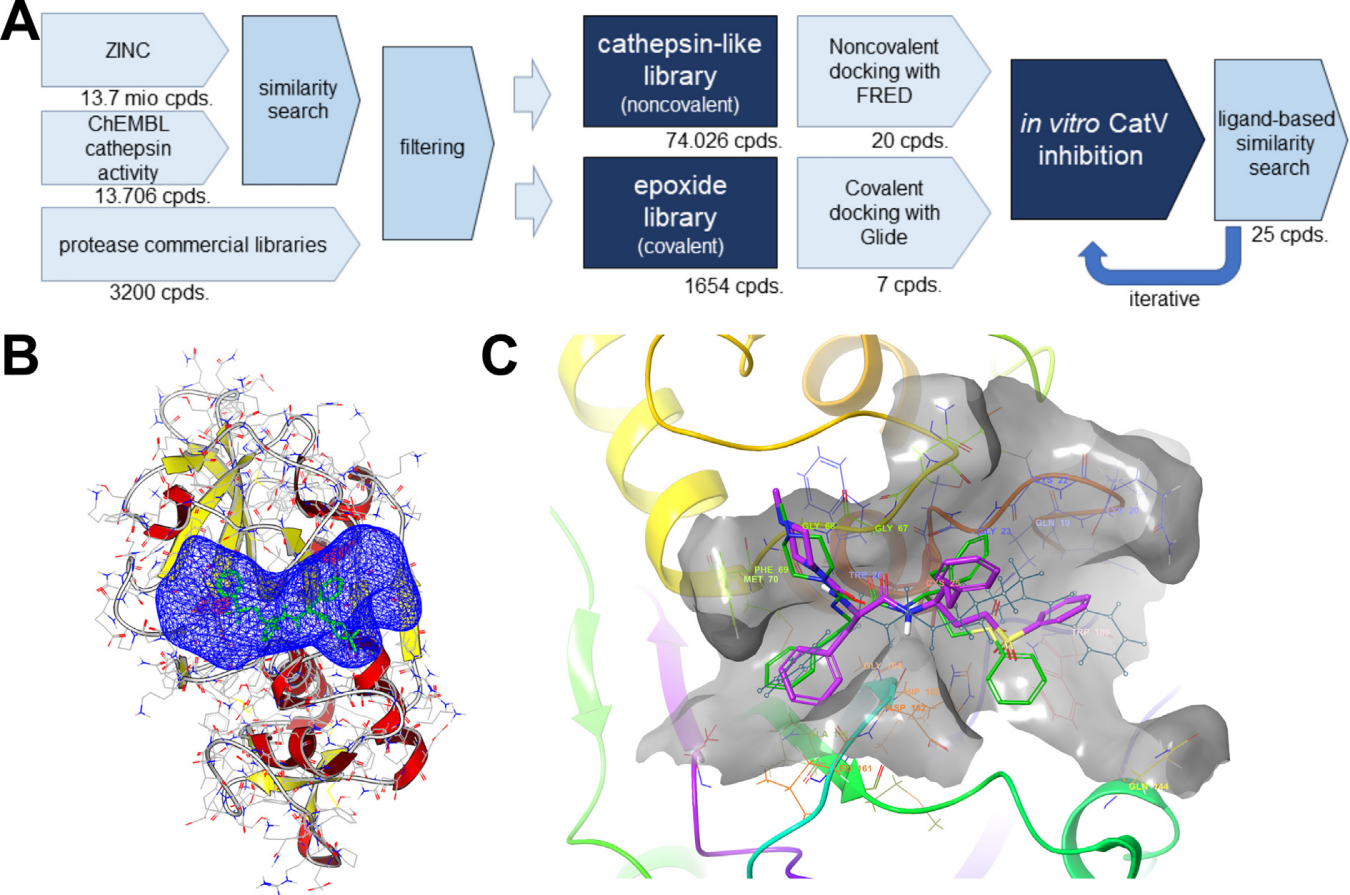
(A) Flowchart representing the process of virtual screening. (B) Identification of the active site in the vicinity of human cathepsin V Cys25 and co-crystalized with the covalent vinyl sulfone inhibitor ligand. The protein (PDB ID: 1FH0) is depicted in a cartoon model with the docking volume highlighted as a blue mesh. (C) The docking protocol for validating the co-crystalized (0IW) covalent vinyl sulfone inhibitor (green stick model) with the docked conformation of 0IW (magenta stick model). The protein is shown in a cartoon model with the residues at the active site (highlighted as lines) and transparent surface (gray). (For interpretation of the references to colour in this figure legend, the reader is referred to the web version of this article.)

First, we used the SQL database ChEMBL to generate an overview of the chemical substances with cathepsin activity (Fig. 1A). Using a SQL query script, we compiled compounds acting on the following human cysteine peptidases: cathepsins B and K (76 compounds), H and K (64), L and K (103), S and K (89), V and K (46), B (3641), F (57), H (176), K (2775), L (3478), L2 (265), S (3101), and X (15). In total, 13,706 compounds made up the library of cathepsin-active chemicals. The ZINC15 *in-stock* subset [39], containing 13.7 million compounds, was used to build a library of compounds similar to cathepsin inhibitors. This expanded library covers a larger chemical space of cysteine peptidases inhibitors and provides the opportunity to identify novel scaffolds. Using OpenBabel (v2.3.0), a similarity search was initiated in the ZINC subset with cathepsin-active queries and a Tanimoto index of ≥ 0.9. FP2 molecular fingerprints were computed for identified cathepsin queries and the ZINC15 *in-stock* subset [40]. The ZINC library was supplemented with targeted or focused libraries from commercial vendors (Life Chemicals Inc., Niagara-on-the-Lake, Canada), such as the peptidase-focused library (designed by Life Chemicals Inc. using a combination of docking and similarity search techniques with ChEMBL reference on compounds with activities of IC_50_ > 1 mM; 1400 compounds) and the cysteine peptidase–focused library (designed by Life Chemicals Inc. using similarity-based techniques, Tanimoto > 0.9 on MDL public keys, with 9618 reference compounds from the literature; 3200 compounds).

Considering the chemical space of the peptidase inhibitors, the similarity library was filtered using the software FILTER, eliminating compounds containing metals and retaining only compounds with appropriate molecular weights and partition coefficients (OpenEye Scientific Software, Inc., Santa Fe, NM, USA; https://www.eyesopen.com; parameters: eliminate metals, allowed elements H, C, N, O, S, F, Cl, Br, max_mw 700, max_logP 9, max_rot_bonds 16). A final library of 50,816 compounds that cover the active space of compounds acting on human cysteine peptidases was obtained. Compounds were ionized in high-throughput virtual screening fashion using FixpKa software (OpenEye Scientific Software; parameter: ionize at neutral pH = 7.4). Finally, OMEGA (OpenEye Scientific Software) was used to enumerate all possible stereocenters (parameter: flipper true) and generate up to 250 conformers per compound (parameter: rms of 0.5; 74,026 molecules were processed with an average of 5 rotors and 160 conformers per compound) [41]. To complement the library with potential covalent inhibitors, an epoxide moiety query (SMARTS: C1[*O*]C1) was employed using obgrep software (OpenBabel; v2.3.0) to construct a filtered list of compounds bearing this moiety. The list was further processed using Ligprep software by Schrödinger (Small molecule discovery suite, Schrödinger, LLC, New York, USA, 2021) with OPLS3 force field, ionizer at pH = 7.4 and no threshold [42]. Tautomery and chirality was determined from input structures to obtain a final epoxide library of 1654 compounds.

### 2.2 Receptor preparation and virtual screening

Studying cathepsin V crystal complexes, we identified a cysteine peptidase in complex with a covalent vinyl sulfone inhibitor (0IW) with a resolution of 1.60 Å, published by Somoza et al. (PDB ID: 1FH0; [5]). The ligand (0IW; *N*alpha-[(4-methylpiperazin-1-yl)carbonyl]-*N*-[(3S)-1-phenyl-5-(phenylsulfonyl)pentan-3-yl]-l-phenylalaninamide) had a Michael acceptor as an electrophilic warhead that reacted with cysteine in the active site. The covalent bond (to Cys25) was cleaved, the 0IW reference ligand removed, and the cysteine amino acid residue regenerated (open source PyMOL, version 2.1; [43]). The structure was cleaned up (to remove SO_4_^2−^), missing hydrogen atoms were added, overlapping atoms were adjusted, hydrogen bonding was optimized, and the ionization of the protein at pH = 7.4 was assigned using Yasara Structure [44], [45]. The postulated binding site was defined around the 0IW ligand near Cys25 Chain A (PDB ID: 1FH0). The docking receptor structure was created using the software package OEDocking 3.2.0.2, Make Receptor Software (OpenEye Scientific Software). A box with a volume of 12333 Å^3^ (24.67 × 20.00 × 25.00 Å) was defined around the cleaved vinyl sulfone ligand (0IW) of chain A, covering the active site (Fig. 1B). Balanced site shape was calculated, with a docking volume of 1966 Å^3^ (no inner site shape restriction was used). No constraints were used, and the conformation or protonation state of the active site residues was not changed. For covalent docking, cleaved crystal complex chain A was prepared with a protein preparation tool (Small molecule discovery suite), H-bonding network was optimized, and minimization was performed (all heavy atoms Root Mean Square Deviation (RMSD; 0.3 Å, OPLS3). The cleaved vinyl sulfone inhibitor was kept as a reference ligand.

In the final step, a prepared cathepsin active chemical space library with an average of 160 conformers per compound was used for the virtual screening experiment. The compounds were docked to the prepared receptor using FRED 3.0.1 (OpenEye Scientific Software) [46]. We used high-resolution docking parameters and the Chemgauss4 scoring function [47], [48]. Validation was performed by re-docking the receptor crystal complex vinyl sulfone ligand (0IW) onto the prepared docking receptor. FRED 3.0.1 successfully reproduced the experimentally determined inhibitor conformation (PDB entry: 1FH0) with an RMSD value of 2.71 Å (Fig. 1C). Finally, the prepared epoxide library was covalently docked (virtual screening, enrichment mode) into the receptor using Schrödinger Glide (Small molecule discovery suite) [49]. The docking volume was defined as a centroid of a cleaved 0IW workspace ligand with inner box size of 10 × 10 × 10 Å, similar ligands to reference, center: 51.430122, 50.003488, 25.839341, outer box size: 26.466682, 26.466682, 26.466682, init_gscore_cutoff: 2.5, max_init_poses: 200, output_top: 200 and 1 output pose parameters. The attachment residue was Cys25 on chain A, and the reaction was defined as epoxide opening (SMARTS: [C;r3][O;r3][C;r3]; position: 1). No core, positional, H-Bond, metal, or torsional constraints were used in the covalent docking experiment.

### 2.3 Similarity search

The ZINC *in-stock* subset library [39] was further employed for similarity search on docking hit compound queries. FP2 molecular fingerprints were calculated for the complete ZINC subset. Using OpenBabel (v2.3.0), a similarity search was initiated in the ZINC subset with hit compound queries and a Tanimoto index of ≥ 0.90. Compounds were filtered based on immediate commercial availability and analytics availability from commercial vendors.

### 2.4 Compound characterization

Compounds were obtained from various sources (Life Chemicals, ENAMINE, Vitas-M, ChemDiv, UkrOrgSynthesis, Otava ltd in InterBioScreen) and used as received. The identity of compounds was confirmed with nuclear magnetic resonance, recorded on a Bruker Avance III 400 MHz spectrometer, using DMSO‑*d*_6_ as a solvent. Chemical shifts are reported in *parts per million* (ppm), and the central peak of the residual solvent resonance at 2.50 ppm was used as the reference. The multiplicities are reported as follows: *s* (singlet), *d* (doublet), *t* (triplet), *q* (quartet), *m* (multiplet), *dd* (doublet of doublets), *ddd* (doublet doublet of doublets), *tt* (triplet of triplets), *dt* (doublet of triplets), *td* (triplet of doublets), *qd* (quartet of doublets), and *br* (broad), coupling constants (*J*) quoted in Hertz (Hz), number of equivalent nuclei (by integration). To further confirm the identity of compounds, high-resolution mass spectrometry measurements were performed on a Thermo Scientific Q Exactive Plus Hybrid Quadrupole-Orbitrap mass spectrometer (Thermo Fisher Scientific, Waltham, MA, USA). Compound purity was determined by HPLC analysis on Thermo Scientific Dionex UltiMate 3000 modular system (Thermo Fisher Scientific Inc.) with Waters Acquity UPLC® HSS C18 SB column (2.1 × 50 mm, 1.8 µm) thermostated at 40 °C, injection volume, 1 µL; flow rate, 0.3 mL/min; detector λ, 220 nm and 254 nm; mobile phase A: 0.1 % TFA (v/v) in water; mobile phase B: MeCN. Method: 0–9 min, 5 %–95 % B; 9–11 min, 95 % B; 11–11.5 min, 95 %-5% B. The best-performing inhibitors are > 95 % pure by HPLC analysis, unless stated otherwise.

#### 2.4.1 *N*-(3-acetylphenyl)-5-oxo-5*H*-thiazolo[3,2-*a*]pyrimidine-6-carboxamide (**4**)

^1^H NMR (400 MHz, DMSO‑*d*_6_) *δ* 11.13 (s, 1H), 8.91 (s, 1H), 8.34 (d, *J* = 4.8 Hz, 1H), 8.31 (t, *J* = 1.9 Hz, 1H), 7.95 (ddd, *J* = 8.1, 2.2, 1.0 Hz, 1H), 7.84 (d, *J* = 4.8 Hz, 1H), 7.73 (dt, *J* = 7.8, 1.3 Hz, 1H), 7.54 (t, *J* = 7.9 Hz, 1H), 2.60 (s, 3H). HRMS (ESI^+^) *m*/*z* [M + H]^+^, calcd. for C_15_H_12_N_3_O_3_S: 314.05939, found: 314.05862. Purity by HPLC: 100 % (tr = 5.107 min, 254 nm).

#### 2.4.2 *N*-(5-benzyl-4,5,6,7-tetrahydrothiazolo[5,4-*c*]pyridin-2-yl)-1-naphthamide (**5**)

^1^H NMR (400 MHz, DMSO‑*d*_6_) *δ* 12.86 (s, 1H), 8.23 – 8.16 (m, 1H), 8.14 (d, *J* = 8.3 Hz, 1H), 8.10 – 8.00 (m, 1H), 7.85 (dd, *J* = 7.1, 1.2 Hz, 1H), 7.63 (tdd, *J* = 7.2, 4.7, 2.5 Hz, 5H), 7.51 (p, *J* = 3.4 Hz, 3H), 4.58 – 4.47 (m, 2H), 4.47 – 4.32 (m, 2H), 3.79 – 3.68 (m, 1H), 3.47 – 3.42 (m, 1H), 3.15 – 2.93 (m, 2H). HRMS (ESI^+^) *m*/*z* [M + H]^+^, calcd. for C_24_H_22_N_3_OS: 400.14781, found: 400.14668. Purity by HPLC: 92 % (tr = 5.177 min, 254 nm).

#### 2.4.3 Phenyl 4-((3-(naphthalen-1-ylmethyl)ureido)methyl)piperidine-1-carboxylate (**7**)

^1^H NMR (400 MHz, DMSO‑*d*_6_) *δ* 8.13 – 8.06 (m, 1H), 7.98 – 7.89 (m, 1H), 7.83 (dt, *J* = 7.7, 1.2 Hz, 1H), 7.60 – 7.33 (m, 6H), 7.25 – 7.16 (m, 1H), 7.13 – 7.05 (m, 2H), 6.31 (t, *J* = 5.7 Hz, 1H), 6.03 (t, *J* = 5.9 Hz, 1H), 4.68 (d, *J* = 5.7 Hz, 2H), 4.15 (d, *J* = 13.3 Hz, 1H), 4.01 (d, *J* = 13.1 Hz, 1H), 2.99 (t, *J* = 6.1 Hz, 3H), 2.88 – 2.76 (m, 1H), 1.71 – 1.56 (m, 3H), 1.14 – 1.10 (m, 2H). HRMS (ESI^+^) *m*/*z* [M + H]^+^, calcd. for C_25_H_28_N_3_O_3_: 418.21252, found: 418.21128. Purity by HPLC: 100 % (tr = 6.430 min, 254 nm).

#### 2.4.4 Methyl ((2*S*,3*S*)-3-(propylcarbamoyl)oxirane-2-carbonyl)-*l*-isoleucyl-*d*-prolinate (**25**)

^1^H NMR (400 MHz, DMSO‑*d*_6_) *δ* 8.76 (d, *J* = 8.4 Hz, 1H), 8.34 (t, *J* = 5.8 Hz, 1H), 4.42 (t, *J* = 8.7 Hz, 1H), 4.33 (dd, *J* = 8.5, 5.3 Hz, 1H), 3.77 (dt, *J* = 9.9, 6.7 Hz, 1H), 3.65 (d, *J* = 1.8 Hz, 1H), 3.61 (s, 3H), 3.66 – 3.56 (m, 1H), 3.47 (d, *J* = 1.9 Hz, 1H), 3.04 (tt, *J* = 7.0, 5.5 Hz, 2H), 2.17 (ddt, *J* = 12.1, 8.6, 6.8 Hz, 1H), 1.98 – 1.73 (m, 3H), 1.55 – 1.35 (m, 2H), 1.11 (ddd, *J* = 13.3, 9.1, 7.0 Hz, 1H), 0.91 (d, *J* = 6.7 Hz, 3H), 0.83 (td, *J* = 7.4, 1.6 Hz, 6H). HRMS (ESI^+^) *m*/*z* [M + H]^+^, calcd. for C_19_H_32_N_3_O_6_: 398.22856, found: 398.22696. Purity by HPLC: 95 % (tr = 4.537 min, 220 nm).

#### 2.4.5 (3-(Benzofuran-2-yl)oxiran-2-yl)(2-(benzyloxy)-4,5-dimethylphenyl)methanone (**27**)

^1^H NMR (400 MHz, DMSO‑*d*_6_) *δ* 7.62 (ddd, *J* = 7.6, 1.5, 0.7 Hz, 1H), 7.52 – 7.44 (m, 2H), 7.33 (ddd, *J* = 8.3, 7.2, 1.4 Hz, 1H), 7.30 – 7.20 (m, 3H), 7.13 (s, 1H), 7.09 – 7.01 (m, 3H), 7.02 (d, *J* = 0.9 Hz, 1H), 5.12 – 5.01 (m, 2H), 4.94 (d, *J* = 2.1 Hz, 1H), 4.31 (d, *J* = 2.0 Hz, 1H), 2.26 (s, 3H), 2.19 (s, 3H). HRMS (ESI^+^) *m*/*z* [M + H]^+^, calcd. for C_26_H_23_O_4_: 399.15909, found: 399.15825. Purity by HPLC was not determined due to instability.

#### 2.4.6 (2-(Benzyloxy)phenyl)(3-(2-phenylthiazol-4-yl)oxiran-2-yl)methanone (**40**)

^1^H NMR (400 MHz, DMSO‑*d*_6_) *δ* 7.94 – 7.85 (m, 2H), 7.84 (s, 1H), 7.66 (dd, *J* = 7.7, 1.8 Hz, 1H), 7.57 (ddd, *J* = 8.8, 7.3, 1.8 Hz, 1H), 7.53 – 7.43 (m, 3H), 7.31 (dt, *J* = 5.8, 3.5 Hz, 2H), 7.24 (d, *J* = 8.4 Hz, 1H), 7.22 – 7.12 (m, 3H), 7.08 (t, *J* = 7.5 Hz, 1H), 5.16 – 5.04 (m, 2H), 4.97 (d, *J* = 2.0 Hz, 1H), 4.27 (d, *J* = 2.0 Hz, 1H). HRMS (ESI^+^) *m*/*z* [M + H]^+^, calcd. for C_25_H_20_NO_3_S: 414.11584, found: 414.11471. Purity by HPLC: 90 % (tr = 7.507 min, 254 nm).

#### 2.4.7 Phenyl 4-((3-benzhydrylureido)methyl)piperidine-1-carboxylate (**42**)

^1^H NMR (400 MHz, DMSO‑*d*_6_) *δ* 7.37 (t, *J* = 7.8 Hz, 2H), 7.36 – 7.28 (m, 4H), 7.28 – 7.16 (m, 7H), 7.13 – 7.06 (m, 2H), 6.84 (d, *J* = 8.5 Hz, 1H), 6.05 (t, *J* = 5.9 Hz, 1H), 5.89 (d, *J* = 8.5 Hz, 1H), 4.14 (d, *J* = 13.3 Hz, 1H), 4.00 (d, *J* = 13.2 Hz, 1H), 3.00 – 2.92 (m, 3H), 2.87 – 2.75 (m, 1H), 2.55 (s, 1H), 1.66 (d, *J* = 13.7 Hz, 2H), 1.11 (s, 2H). HRMS (ESI^+^) *m*/*z* [M + H]^+^, calcd. for C_27_H_30_N_3_O_3_: 444.22817, found: 444.22702. Purity by HPLC: 96 % (tr = 6.547 min, 254 nm).

### 2.5 Permeability calculation

The permeability of compound **7** was predicted using QikPrep software from Schrödinger (Small molecule discovery suite, Schrödinger, LLC, New York, USA, 2021). Compound was first processed using LigPrep to generate appropriate tautomeric and ionization states at pH 7.0. Next, QikProp was run with the default settings to predict the apparent Caco-2 cell permeability, which is a model for the gut–blood barrier (< 25 nm/s poor, > 500 nm/s great), and the apparent Madin − Darby Canine Kidney cells (MDCK) cell permeability, which is a model for the blood–brain barrier (< 25 nm/s poor, > 500 nm/s great).

### 2.6 Aqueous stability

HPLC analyses were performed as described under the Compound characterization section. The method used a Waters Acquity UPLC® HSS C18 SB column (2.1 × 50 mm, 1.8 µm) thermostated at 40 °C, injection volume, 10 µL; flow rate, 0.3 mL/min; detector λ, 280 nm; mobile phase A: 0.1 % TFA (v/v) in water; mobile phase B: MeCN. Method: 0–9 min, 5 % – 95 % B; 9 – 11 min, 95 % B; 11 – 11.5 min, 95 % - 5% B.

Compound **7** stock solution (1 mM) was prepared in DMSO, while internal standard (caffeine, 1 mM) was dissolved in buffer (PBS pH 7.4). The aqueous stability solution was prepared by mixing 1400 µL buffer, 400 µL MeCN, 100 µL internal standard stock solution, and 100 µL compound stock solution in a HPLC vial to give 50 μM compound, 50 μM internal standard, 20 % MeCN, and 5 % DMSO in buffer, which was injected immediately and then at hourly intervals for 60 h while being incubated at 37 °C. The analyte areas under curve (AUCs) were normalized on internal standard AUCs.

### 2.7 Kinetic solubility

The solubility of compound **7** was determined in buffer (PBS pH 7.4) containing 5 % DMSO. Serial dilutions of 7 were prepared in DMSO at concentrations of 4 mM, 2 mM, 1.5 mM, 1 mM, and 0.2 mM. The assay was performed in 96-well microplates in duplicate. Briefly, to 190 µL of buffer, 10 µL of the compound stock solution in DMSO was added. The plate was shaken and the absorbance at 620 nm was measured using a plate reader (Synergy H4, BioTek Instruments, Inc., Santa Clara, CA, USA) in area scan mode (5 × 5 measurements per well). The mean absorbance was then compared relative to the blank to determine the solubility of the compound at each concentration tested.

### 2.8 Enzyme kinetics

Human recombinant cathepsin V was expressed in *Pichia Pastoris*, and human recombinant cathepsin L was expressed in *Escherichia coli*, as reported [50], [51]. To determine the activities of cathepsins V and L, 100 mM acetate buffer (pH 5.5), containing 0.1 % PEG 8000 (Sigma-Aldrich, St. Louis, MO, USA), 5 mM cysteine, and 1.5 mM EDTA was used. Prior to the assay, the enzyme was activated in the assay buffer for 5 min at 37 °C.

#### 2.8.1 Determination of relative inhibition

The effect of the inhibitors on cathepsin V and L activity was determined using the substrate Z-Phe-Arg-AMC (Bachem, Bubendorf, Switzerland). The reaction was initiated by adding 90 μL of the activated enzyme (final concentration 2.5 ng/mL and 0.02 nM for cathepsins V and L, respectively) to the wells of a black microtiter plate containing 5 μL of substrate (with a final concentration 2 μM) and 5 μL of inhibitor (with a final concentration of 50 μM). The formation of fluorescent degradation products during the reaction was monitored continuously at 460 nm ± 10 nm with excitation at 380 nm ± 20 nm at 37 °C on a Tecan Infinite M1000 (Mannedorf, Switzerland) spectrofluorometer. All assay mixtures contained 5 % (v/v) DMSO. To prevent false-positive inhibition due to the formation of compound aggregates, 0.01 % Triton X-100 was used [52]. All measurements were performed in triplicate and repeated at least twice. Relative inhibition was calculated using the following equation: *relative inhibition (%) = 100(1- v_i_/v_o_)*, where *v_i_* and *v_o_* denote the reaction velocities in the presence and absence of the inhibitor, respectively.

#### 2.8.2 Determination of Ki values

Inhibition constants were calculated from reaction velocities measured at three substrate concentrations (0.5, 1, and 4 μM) in the presence of inhibitors at seven concentrations (0, 20, 40, 60, 80, 100, and 200 µM). The reaction was initiated by adding 90 μL of enzyme at final concentrations of 2.5 ng/mL and 0.02 nM for cathepsins V and L, respectively, to the wells of a black microtiter plate containing 5 μL of substrate and 5 μL of inhibitor. To evaluate cathepsin V and L activity, the substrate Z-Phe-Arg-AMC was used. The formation of the fluorescent degradation products was monitored as described above. All assay mixtures contained 5 % (v/v) DMSO. All measurements were performed in duplicates and repeated at least twice. The SigmaPlot® 12, Enzyme Kinetics Module™ 1.3 was used to calculate K_i_ values.

### 2.9 Determination of binding reversibility

Binding reversibility was determined according to the dilution method, which measures the recovery of enzymatic activity after rapid dilution of the enzyme-inhibitor complex. Cathepsin V (500 ng/ml) was incubated in the assay buffer with the inhibitor (100 μM). Half of the reaction mixture was immediately diluted 100-fold with the assay buffer and added to the wells of a black microplate containing 5 μL of substrate (at a final concentration of 2 μM), and the activity was measured. The other half of the reaction mixture was incubated for 2 h before it was diluted 100-fold, and then the activity was measured. The assay was performed in triplicates and repeated at least two times.

### 2.10 Cell lines

The human epithelial mammary gland adenocarcinoma cell line MCF7 was cultured in Dulbecco’s modified Eagle’s medium (DMEM)/Nutrient Mixture F-12 (1:1) medium with GlutaMAX™ (Gibco, Carlsbad, CA, USA) supplemented with 5 % fetal bovine serum (FBS, Gibco), 1 µg/mL insulin (Sigma-Aldrich), 0.5 µg/mL hydrocortisone (Sigma-Aldrich), 20 ng/mL epithelial growth factor (Milipore, Burlington, MA, USA) and 1 % penicillin–streptomycin (Gibco), corresponding to 100 U/mL penicillin and 100 µg/mL streptomycin. Human promonocyte cell line U-937 and lymphoblast cell line K-562 were cultured in RPMI 1640 medium (Lonza, Basel, Switzerland) supplemented with 10 % heat-inactivated FBS (Gibco) and 1 % penicillin–streptomycin (Gibco). NK-92 cells (NK cell line) were cultured in RPMI 1640 media supplemented with 12.5 % heat-inactivated FBS, 12.5 % heat-inactivated horse serum (Gibco), 1 % penicillin/streptomycin, and 200 IU IL-2/mL (Bachem). TALL-104 cells (CTL cell line) were cultured in RPMI 1640 media (ATCC, Manassas, VA, USA) containing 20 % heat-inactivated FBS and 150 IU IL-2/mL. All cell lines were obtained from the American Type Culture Collection (ATCC). All cell lines had tested negative for mycoplasma (Mycoplasmacheck, Eurofins Genomics, Ebersberg, Germany). Cells were maintained and cultured at 37 °C in a humidified atmosphere containing 5 % CO_2_. Prior to use, adherent cells were detached from culture flasks using TripLE™ Select Enzyme (Gibco). U-937 cells were differentiated to macrophages by treatment with 50 mM phorbol 12-myristate 13-acetate (PMA; Sigma-Aldrich) for 24 h at 37 °C [53]. Thereafter, the medium containing the undifferentiated cells was removed, and only the adherent macrophages obtained from U-937 cells were used for further experiments.

### 2.11 Control inhibitors

In addition to the new cathepsin V inhibitors identified in this study, the following well-established cathepsin inhibitors were used as controls: E-64 and its cell-permeable derivative E-64d (both Sigma-Aldrich) as general cysteine cathepsin inhibitors and CLIK-148, morpholinurea-leucine-homophenylalanine-vinylsulfone-phenyl (LHVS), and pepstatin (Peptide Institute Inc, Osaka, Japan) [54] for cathepsins L, S, and D, respectively. The inhibitor concentrations used in the assays were selected according to the literature for CLIK-148 [55] and LHVS [56] or used at the same concentrations as our new inhibitors. CLIK-148 and LHVS were kindly provided by prof. Nobuhiko Katunuma (University of Tokushima, Japan) and dr. James MeKerrow (University of California, San Francisco, USA), respectively.

### 2.12 Proliferation assay

To assess the effect of cathepsin V inhibitors on cell proliferation, MCF7 cells were fluorescently labeled with carboxyfluorescein succinimidyl ester (CFSE; ImmunoChemistry Technologies, Bloomington, MN, USA) in PBS for 15 min at room temperature according to the manufacturer’s instructions. Cells were washed with PBS, resuspended in complete medium, seeded (at 4 × 10^4^ cells/well) into 24-well plates, and allowed to adhere overnight. Cells were then treated with the following inhibitors: compound **7** (10 μM), E-64d (10 μM), CLIK-148 (1 μM), LHVS (10 nM), pepstatin (20 μM), their combinations, or DMSO (0.1 %) in 500 μL of medium for 72 h at 37 °C. To prepare cells for flow cytometry, cells were detached and transferred to round-bottomed polystyrene tubes and washed with PBS. Before measurement, 7-aminoactinomycin D (7-AAD; ImmunoChemistry Technologies, Bloomington, MN, USA) was added according to the manufacturer’s instructions to exclude dead cells. Therefore, the green fluorescence arising from CFSE was monitored only for viable cells. The measurement was performed using a FACSCalibur instrument (BD Biosciences, San Jose, CA, USA).

### 2.13 Intracellular elastin-FITC degradation

To evaluate the effect of the inhibitors on the elastolytic activity of cathepsin V, we assessed the intracellular degradation of elastin-FITC, a soluble bovine neck ligament elastin that is heavily labeled with FITC such that the fluorescence of the conjugate is quenched. Upon proteolysis, fluorescence is recovered and can be detected. U-937 cells, differentiated into macrophage-like cells, were seeded (at 1 × 10^5^ cells/well) into 24-well plates with 500 μL of medium supplemented with 50 nM PMA. Inhibitors or DMSO (0.1 %) and elastin-FITC (10 μg/mL; AnaSpec, Fermont, CA, USA) were added to the medium and incubated for 24 h at 37 °C. Cells were detached and washed with PBS, and propidium iodide (BD Bioscience) was added to exclude dead cells. Therefore, green fluorescence arising from elastin-FITC degradation was monitored only for viable cells. The measurement was performed using a FACSCalibur instrument (BD Biosciences).

### 2.14 Cell viability assay

The effect of compound **7** on the viability of NK-92, TALL-104, and K-562 cells was evaluated using the MTS [3-(4,5-dimethylthiazol-2-yl)-5-(3-carboxymetoxyphenyl)-2-(4-sulfophenyl)–2*H*-tetrazolium] colorimetric assay (CellTiter 96 Aqueous One Solution Cell Proliferation Assay, Promega, Madison, WI, USA). NK-92, TALL-104, and K-562 cells were seeded at 2.5 × 10^4^, 1 × 10^5^, and 1 × 10^4^ cells/well, respectively, into a 96-well microplate and treated with inhibitors (2.5, 5, or 10 µM) or DMSO (0.1 %) for 24 h. After incubation, 10 µL of the reagent MTS was added to the wells, and the absorbance of formazan was measured at 492 nm on a Tecan Infinite M1000 (Mannedorf, Switzerland). Cell viability (%) was expressed as the ratio between absorbance in the presence of the compounds and in the presence of DMSO. All assays were performed in quadruplicate and repeated at least twice.

### 2.15 Western blot analysis

Cells were treated with E-64d or compound **7** (both at 10 µM or 20 µM) for 6 h. After treatment, whole-cell lysates were prepared in RIPA buffer supplemented with cOmplete™ Protease Inhibitor Cocktail (Roche, Basel, Switzerland) and centrifuged at 16 000 × g for 20 min at 4 °C. Total protein concentration was determined using the DC assay (Biorad, Hercules, CA, USA). Proteins were separated by non-reducing SDS-PAGE and transferred to a nitrocellulose membrane using the TransBlot Turbo System (Biorad). After blocking, the membranes were incubated with primary antibodies overnight at 4 °C and with secondary antibodies for 1 h at room temperature. Bands were visualized using Clarity Max Western ECL substrate (Biorad), and protein loading was checked using Stain Free technology (Biorad). Images were acquired using the ChemiDoc ML Imaging System (Biorad), and quantification analysis was performed using Image Lab software (Biorad).

The following primary antibodies were used: rabbit anti-cystatin F (1:375, HPA040442, Sigma), goat anti-cathepsin V (1:200, AF 1080, R&D, USA), and mouse anti-GAPDH (1:3000, 10494-I-AP, Proteintech, USA). The following secondary antibodies were used: anti-rabbit (1:5000, 111–035-045, Jackson ImmunoResearch, Baltimore, PA, USA) and anti-goat (1:5000, sc2354, Santa Cruz Biotechnology, Dallas, TX, USA) conjugated with horseradish peroxidase and anti-mouse StarBright 700 (1:5000, 12004158, Biorad).

### 2.16 Calcein AM release assay

Effector cells (NK-92 or TALL-104; 1 × 10^6^/ml) were treated with E-64d (10 µM) or compound **7** (10 µM) for 6 h. NK-92 cells were pre-treated with 1000 IU IL2/mL overnight prior to the addition of inhibitors. Effector cells were added to a 96-well U-bottom plate to achieve a selected effector-to-target ratio (E:T). Target K-562 cells were labelled with 15 µM Calcein-AM (Sigma) in serum-free RPMI 1640 medium for 30 min at 37 °C. Cells were then washed twice and resuspended in complete RPMI 1640 medium. Target cells (n = 5000) were added to the effectors. The plate was centrifuged at 200 × g for 1 min and incubated at 37 °C and 5 % CO_2_ for 4 h. After incubation, the plate was centrifuged at 700 × g for 5 min. The supernatant (50 µL) was transferred to a new microtiter plate to measure the fluorescence of the released Calcein-AM with a Tecan M1000 microtiter plate reader using 496 nm excitation and 516 nm emission filters. The percentage of cytotoxicity was calculated as follows: *100(test release-spontaneous release)/(total release-spontaneous release)*. Spontaneous release was measured in wells containing only target cells. For total release, 2 % Triton X-100 was added to the wells to achieve lysis of target cells. Lytic units (LU) were calculated using the inverse of the number of effector cells needed to lyse 30 % of the target cells multiplied by 100.

### 2.17 Statistical analysis

The GraphPad Prism 9.0 software package (GraphPad Software, San Diego, USA) was used for data analysis. Data are presented as means ± SEM unless stated otherwise. Statistically significant differences between data groups were assessed using the nonparametric, two-tailed Student’s *t*-test or one-way analysis of variance (ANOVA). Differences were considered significant at *P* ≤ 0.05.

## 3 Results

### 3.1 Molecular docking

Virtual screening was performed in two separate experiments, namely noncovalent docking of the cathepsin library using Fred software (OpenEye Scientific Software) and covalent docking of the epoxide library with Glide from Schrödinger (Small molecule discovery suite) to the prepared crystal structure of human cathepsin V complexed with an irreversible vinyl sulfone inhibitor (PDB ID: 1FH0). The top 20 hit compounds **1**–**20** (Table 1) from the noncovalent docking experiment were acquired for biological evaluation. The hit-list was supplemented with seven commercially available epoxides as potential covalent binders, **21**–**27** (Table 1), from the second docking experiment.

**Table 1:** Inhibitory activity of structure-based series of compounds selected as potential cathepsin V inhibitors.

		**Docking score^a^**		**Cathepsin V**			**Cathepsin L**		
**Compound**		**Fred**	**Glide**	**Ri (%)^b^**	**Ki (µM)^c^**	**Ki′ (µM)^c^**	**Ri (%)^b^**	**Ki (µM)^c^**	**Ki′ (µM)^c^**
**1**		−10.5		−5.4 ± 2.4			4.8 ± 3.9		
**2**		−11.6		16.4 ± 0.3			20.0 ± 2.2	162.3 ± 1.8^d^	
**3**		−11.4		382.0 ± 13.3			146.6 ± 88.7		
**4**		−10.7		23.9 ± 2.9	352.2 ± 78.6^d^		23.5 ± 2.2	84.3 ± 4.0^d^	
**5**		−9.6		−5.8 ± 7.0			30.3 ± 5.5		11.2 ± 0.1^e^
**6**		−9.5		33.5 ± 0.7	371.3 ± 86.1^d^		40.3 ± 4.5	635.2 ± 166.7^d^	
**7**		−11.8		24.0 ± 15.6	88.7 ± 10.2^d^		12.1 ± 1.8	n.i^f^	
**8**		−8.8		−20.3 ± 3.5			−4.4 ± 2.1		
**9**		−10.2		18.9 ± 1.3			17.5 ± 5.3		
**10**		−10.9		17.8 ± 3.3			21.5 ± 1.2		
**11**		−11.7		−3.1 ± 4.6			6.3 ± 2.6		
**12**		−9.3		−9.5 ± 9.2			15.6 ± 1.2		
**13**		−9.6		7.5 ± 5.0			13.4 ± 1.4		
**14**		−9.0		7.8 ± 3.1			13.1 ± 2.6		
**15**		−9.1		−27.6 ± 9.1			−5.4 ± 7.3		
**16**		−9.0		−6.5 ± 0.9			−1.0 ± 4.8		
**17**		−10.5		−2.4 ± 5.6			9.4 ± 9.5		
**18**		−9.2		6.7 ± 0.03			13.0 ± 7.9		
**19**		−9.3		3.8 ± 3.5			17.8 ± 13.7		
**20**		−8.7		16.9 ± 0.6			14.5 ± 2.4		
**21**			−6.8	25.3 ± 4.1	159.3 ± 32.8^d^		15.3 ± 2.9	235.8 ± 10.3^d^	
**22**			−6.8	−7.2 ± 7.7			11.1 ± 4.4		
**23**			−6.7	−2.9 ± 1.8			11.9 ± 6.2		
**24**			−6.7	0.8 ± 3.3			17.7 ± 11.7		
**25**			−6.7	83.1 ± 6.8	8.0 ± 0.02^d^		83.7 ± 1.0	9.9 ± 0.9^d^	
**26**			−7.1	6.3 ± 4.6			10.6 ± 4.5	375.0 ± 20.5^d^	
**27**			−7.0	31.7 ± 5.1	68.3 ± 4.6 ^g^		22.6 ± 5.1	248.6 ± 29.8^d^	

^a^Docking scores in kcal/mol. ^b^ Relative inhibition (Ri) determined at 50 μM, values are the mean ± SD (n = 2). ^c^ Ki and Ki′ values are the mean ± SD (n = 2). ^d^ Competitive inhibition. ^e^ Uncompetitive inhibition. ^f^ n.i. – no inhibition. ^g^ Noncompetitive inhibition.

**Fig. 2:**
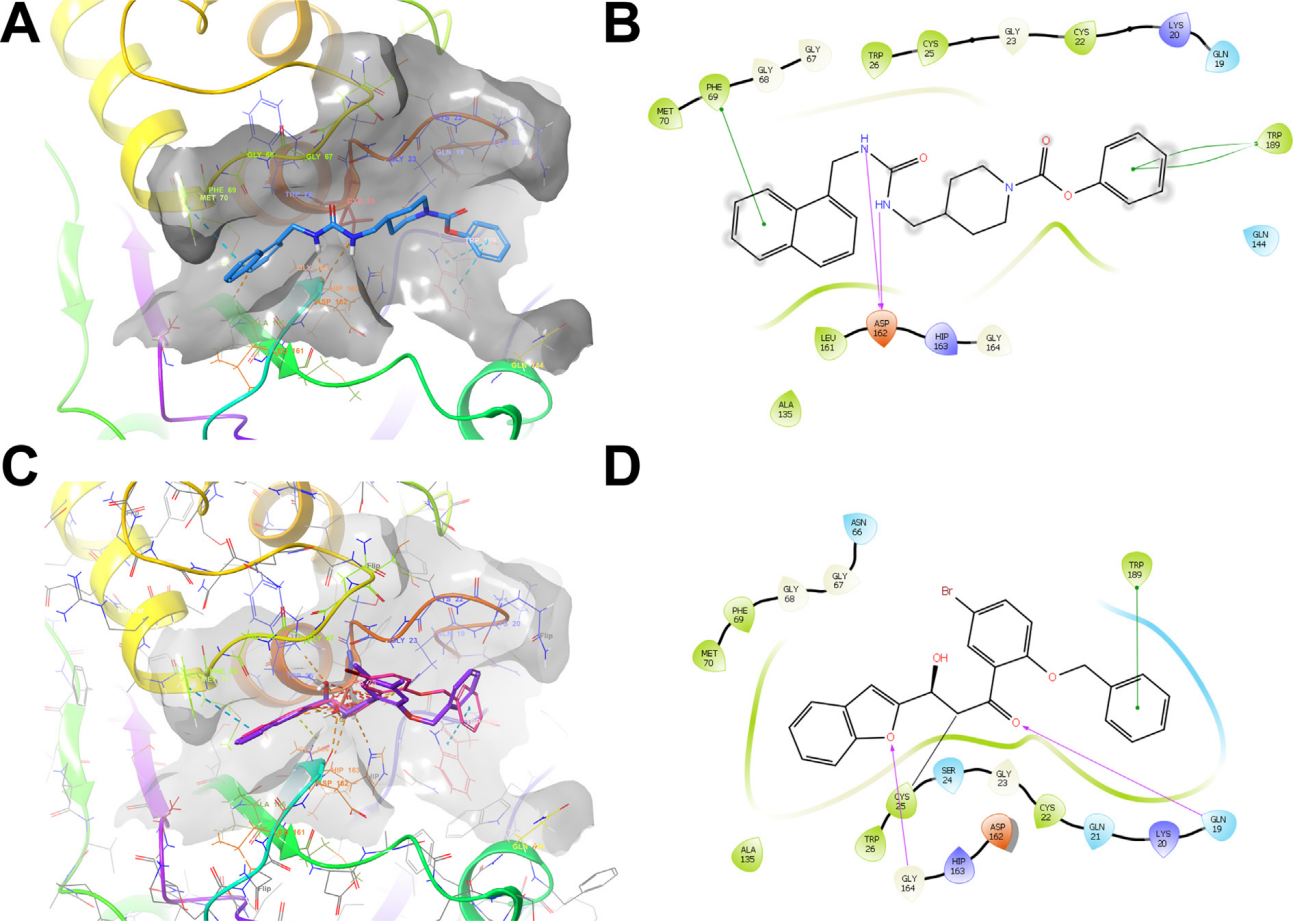
Calculated binding modes of noncovalent and covalent hit compounds. (A) Calculated binding mode of compound **7** (blue stick model) at the active site of cathepsin V (PDB ID: 1FH0). The surface of the protein (grey), catalytic Cys25 (red rods), and the contacts of the compounds (dotted lines) are shown. (B) Interaction diagram of compound **7**, 2D projection. (C) Calculated covalent binding mode of the highest scoring compounds, **26** (pink sticks) and **27** (magenta sticks). The active site of cathepsin V (PDB ID: 1FH0) (line model with grey surface), catalytic Cys25 (grey rod model), and the contacts of the compounds (dotted lines) are shown. (D) Interaction diagram of compound **26**, 2D projection. (For interpretation of the references to colour in this figure legend, the reader is referred to the web version of this article.)

Inspection of the calculated poses of the noncovalent hit compounds revealed a common binding motif in which all docked compounds occupy either one or both cathepsin V binding pockets: S1′ (defined by Gln19, Cys22, Gly23, Cys25, Ala138, Gly139, Asp162, His163, Gly164, and Trp189) and S2 (defined by Gly68, Phe69, Met70, Ala135, Leu161, Asp162, His163, Gly164, and Ala214). The binding modes are exemplified by the top hit compound **7** (see below), where the compound enters into a π-π stacking interaction at the cathepsin V S1′ pocket between the terminal phenyl moiety and Trp189 (spacing 3.79 Å, Fig. 2A). On the opposite side, compound **7** takes advantage of the hydrophobic pocket S2 by making additional π-π and hydrophobic contacts from its naphthyl moiety to Phe69 (4.89 Å) and Ala135 (3.12 Å). The compound is centrally anchored near the key Asp162 with two H-bonds (1.7 Å) via its central urea linker moiety and places its amide mimic 4.2 Å proximal to catalytic Cys25 (Fig. 2B). All other hit compounds are generally positioned analogously by harboring their linkers (urea, amide, or sulfonamide) near the central Asp162 and Cys25 residues and occupying S1′-S2 or individual binding pockets with their terminal lipophilic (halogen-derivatized) moieties.

The calculated binding modes are further supported by a second covalent docking experiment in which the compounds adopt an analogous binding mode, even though they form a covalent bond with catalytic Cys25 via their central electrophilic epoxide moiety, resulting in hydroxythioethers. The highest scoring compounds, **26** and **27** (Fig. 2C), both form π-π stacking interactions in the cathepsin V S1′ pocket between the terminal phenyl groups and Trp189 and additional π-π and hydrogen bonds with their benzofuran groups to Phe69 and Gly164, respectively, in hydrophobic pocket S2 (Fig. 2D). These compounds have the advantage of being close to the catalytic site and form an additional H-bond between the central carbonyl and Gln19. Their central phenyl linker appears to be ideal for future structure active relationship extension and possible expansion of the compounds toward the S3 pocket, and the same can be observed for the linkers of reported noncovalent inhibitors.

### 3.2 Inhibition of cathepsin V and L activity determined by enzyme kinetics

The top hits identified as potential inhibitors by molecular docking were tested for their inhibition of cathepsin V at a concentration of 50 μM. Relative inhibition is expressed as the percentage of decrease in reaction velocity in the presence of the inhibitor compared with the reaction velocity in the absence of the inhibitor. To address the selectivity for cathepsin V, cathepsin L inhibition was determined for all compounds. Relative inhibition relies on the experimental settings including concentration and type of enzyme and substrate, therefore the care must be taken in the interpretation and comparison of results between different enzymes. The substrates used in the reaction are synthetic and not equal to natural ones. The compounds **4**, **6**, **7**, **21**, **25**, and **27** exhibited more than 20 % inhibition of cathepsin V and were selected for further evaluation (Table 1). Compound **3** was not selected for further evaluation due to possible assay interference at the wavelength used to follow the enzyme reaction. Inhibition constants (Ki) and types of inhibition were determined for selected compounds for both cathepsins V and L. Ki values are kinetic parameters, independent of enzyme concentration and were determined at three different substrate concentrations and seven different inhibitor concentrations. Additionally, the inhibition constants and modes of inhibition of cathepsin L were determined for compounds **2** and **5**.

Among the selected compounds, the most potent inhibitors of cathepsin V were compounds **25** (Ki = 8.0 ± 0.02 μM), **27** (Ki = 68.3 ± 4.6 μM), and **7** (Ki = 88.7 ± 10.2 μM), whereas other compounds had higher inhibition constants (Table 1). A competitive mode of inhibition was observed for all compounds tested, indicating that the compounds bind predominantly to the free enzyme, except for compound **27**, which showed a noncompetitive mode of inhibition, binding to the free enzyme and enzyme–substrate complex with the same affinity. Regarding cathepsin L, the lowest inhibition constant was determined for compounds **25** (Ki = 9.9 ± 0.9 μM), **5** (Ki' = 11.2 ± 0.1 μM), and **4** (Ki = 84.3 ± 4.0 μM) (Table 1). Similar to cathepsin V inhibition, a competitive mode of inhibition was observed for cathepsin L inhibition for all compounds tested, including compound **27**. The only exception was compound **5**, which binds predominantly to the enzyme–substrate complex and exhibits an uncompetitive mode of inhibition. The results show that compound **25** is the most potent inhibitor of both enzymes and is not selective for a single cathepsin. Of the other compounds with low inhibitory constants for cathepsin V, compound **7** did not inhibit cathepsin L, and compound **27** had a more than 3-fold lower inhibitory constant for cathepsin V than cathepsin L. Thus, these compounds could be considered specific inhibitors of cathepsin V. Conversely, compounds **4** and **5** did not inhibit cathepsin V and were selective for cathepsin L.

To expand the chemical space of hits obtained from the molecular docking experiments, the structural properties of the best cathepsin V inhibitors were used for another ligand-based similarity search of commercially available compound libraries. An additional 25 structurally related compounds (**28**–**52**) were selected and screened for their inhibition of cathepsin V activity. We first determined their relative inhibition and then their inhibition constants and modes for selected compounds with relative inhibition more than 20 % (Table 2). As the selection of these compounds was based on compounds with selectivity for cathepsin V, lower cathepsin L inhibition was observed. The compounds with the lowest inhibition constant for cathepsin V were compounds **40** (Ki' = 21.2 ± 2.0 μM) and **42** (Ki = 155.8 ± 3.4 μM) (Table 2). Compounds **40** and **42** exhibited uncompetitive and noncompetitive modes of inhibition, respectively. Both compounds **40** and **42** were selective for cathepsin V compared with cathepsin L, with 21-fold and more than 2-fold lower inhibition constants, respectively (Table 2). For compound **31**, the inhibition constant was not determined due to observed test interferences at higher concentrations.

**Table 2:** Inhibitory activity of ligand-based series of compounds selected as potential cathepsin V inhibitors.

		**Cathepsin V**			**Cathepsin L**		
**Compound**		**Ri (%)^a^**	**Ki (µM)^b^**	**Ki′ (µM)^b^**	**Ri (%)^a^**	**Ki (µM)^b^**	**Ki′ (µM)^b^**
**28**		1.1 ± 9.0			−4.6 ± 5.2		
**29**		−0.5 ± 1.2			−2.4 ± 2.0		
**30**		−2.0 ± 3.3			−1.2 ± 0.3		
**31**		29.2 ± 7.0	T.I.^c^		20.9 ± 7.2	T.I.^c^	
**32**		6.9 ± 2.2			4.7 ± 3.2		
**33**		−1.6 ± 6.5			3.4 ± 4.4		
**34**		14,0 ± 10.9			−3.0 ± 0.5		
**35**		16.6 ± 8.4			12.0 ± 1.3		
**36**		15.2 ± 10.0			−0.6 ± 1.7		
**37**		−3.4 ± 2.9			−2.1 ± 0.7		
**38**		7.9 ± 0.4			2.3 ± 3.1		
**39**		17.1 ± 7.2	403.0 ± 79.7^d^		6.1 ± 0.7	725.7 ± 142.3^e^	
**40**		27.6 ± 8.7		21.2 ± 2.0^f^	12.4 ± 0.7	454.0 ± 69.1^d^	
**41**		17.7 ± 6.1	612.5 ± 23.9^e^		4.2 ± 0.9	n.i.^g^	
**42**		21.6 ± 4.6	155.8 ± 3.4^e^		3.5 ± 3.1	319.2 ± 28.6^e^	
**43**		5.2 ± 1.8			−0.4 ± 2.5		
**44**		11.1 ± 11.5			1.1 ± 4.9		
**45**		9.2 ± 6.4			3.3 ± 2.4		
**46**		12.1 ± 9.8			4.3 ± 4.1		
**47**		9.5 ± 2.5			4.4 ± 4.6		
**48**		20.4 ± 14.5	1187.2 ± 468.0^d^		5.7 ± 3.6	1191.2 ± 357.4^d^	
**49**		22.9 ± 1.0	303.0 ± 11.3^d^		12.9 ± 0.5	546.4 ± 114.4^d^	
**50**		−2.9 ± 4.0			13.8 ± 0.5		
**51**		−9.8 ± 5.8			−4.0 ± 7.3		
**52**		0.3 ± 8.8			−0.7 ± 1.5		

^a^Relative inhibition (Ri) determined at 50 μM, values are the mean ± SD (n = 2). ^b^ Ki and Ki′ values are the mean ± SD (n = 2). ^c^ T.I. – test interference, ^d^ Competitive inhibition, ^e^ Noncompetitive inhibition, ^f^ Uncompetitive inhibition, ^g^ n.i. – no inhibition.

### 3.3 Evaluation of binding reversibility

**Fig. 3:**
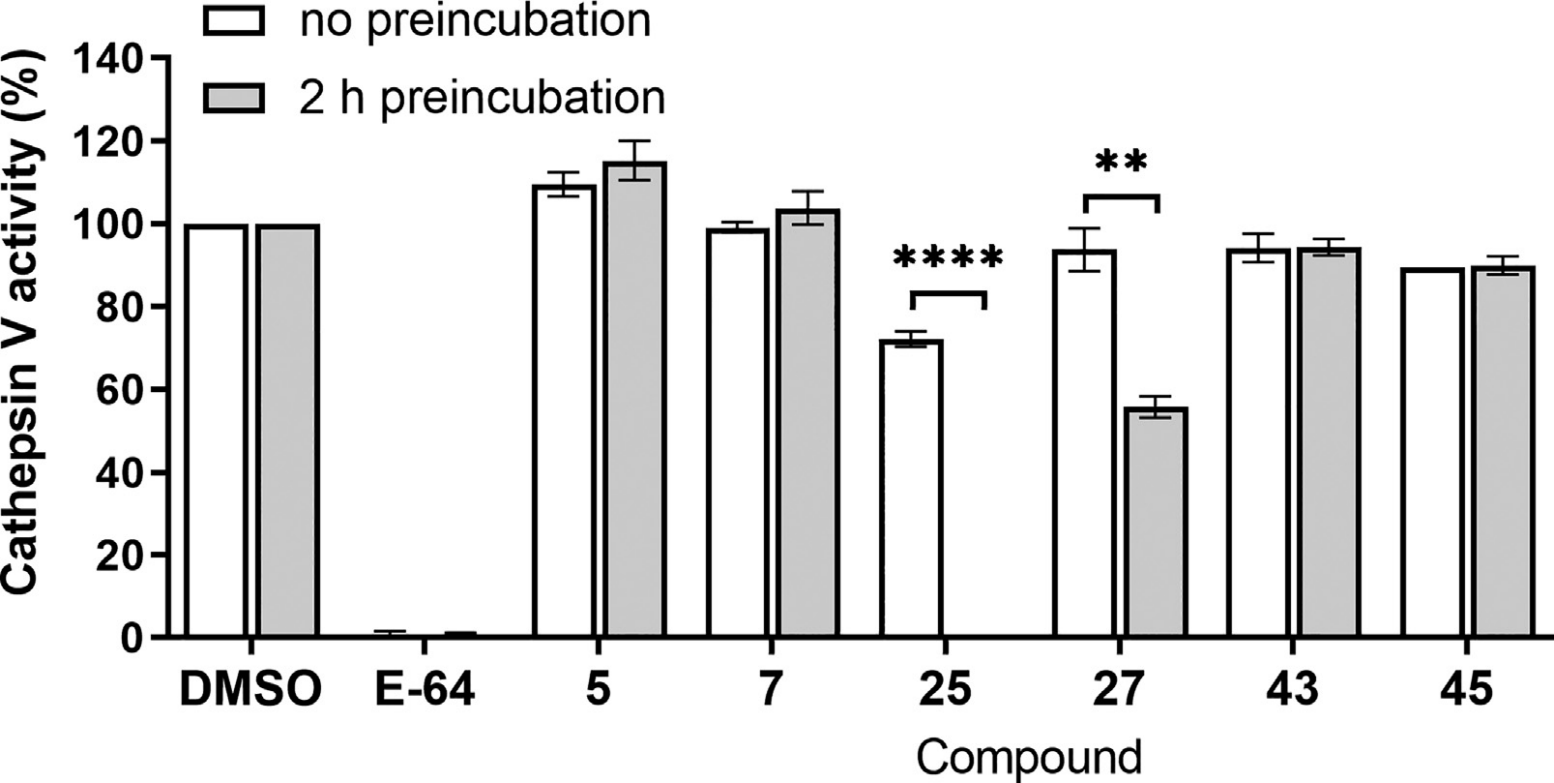
Binding reversibility of selected cathepsin V inhibitors. Cathepsin V activity measured after rapid 100-fold dilution of cathepsin V and inhibitor mixture. The mixture was diluted immediately or after 2 h of incubation. Recovery of enzyme activity after dilution with 2 h incubation points towards reversible binding of the inhibitor. Data are presented as mean ± SEM (n = 2). **P < 0.01, ****P < 0.0001 (Student's *t*-test).

Next, we tested the selected compounds for reversible or irreversible modes of binding with cathepsin V using the dilution method. For reversible inhibitors, rapid dilution of a concentrated enzyme-inhibitor mixture typically recovers enzymatic activity because dilution reduces the concentration of inhibitor in the mixture. Conversely, irreversible inhibitors remain bound to the enzyme, and thus rapid dilution of the mixture does not recover enzymatic activity. In this experiment, we used the general irreversible inhibitor of cysteine cathepsins E-64 as a positive control. As expected for an irreversible inhibitor, the enzyme remained inhibited even after rapid dilution. After 2 h of incubation and 100-fold rapid dilution, the activities of compounds **5**, **7**, **40**, and **42** were restored (Fig. 3), indicating that these compounds are reversible inhibitors [57]. For compound **27**, the activity was only partially restored, and a significant decrease in enzyme activity was observed after 2 h of incubation compared with immediate dilution of the mixture (Fig. 3). As such, this compound could be either a tight-binding or irreversible inhibitor of cathepsin V. By contrast, compound **25** completely blocked cathepsin V after 2 h of incubation and even after rapid dilution (Fig. 3), indicating its irreversible mode of binding.

### 3.4 Permeability calculation, evaluation of aqueous stability and kinetic solubility

Permeability calculation, evaluation of aqueous stability and kinetic solubility were performed for compound **7**, the most potent selective reversible cathepsin V inhibitor, that was used in all *in vitro* cell-based assays. The permeability of compound **7** was predicted using QikPrep software from Schrödinger (Small molecule discovery suite). Both the apparent Caco-2 permeability and the apparent MDCK permeability were classified as “great” with 829 nm/s and 612 nm/s, respectively (< 25 nm/s poor, > 500 nm/s great). Next, aqueous stability for compound **7** was evaluated. The stability data showed that compound **7** is stable for days in the aqueous solution under physiologically similar conditions (Fig. S1). Furthermore, the kinetic solubility of compound **7** was between 75 and 100 μM in PBS with 5 % DMSO.

### 3.5 Cathepsin V inhibitors impair tumor cell proliferation

**Fig. 4:**
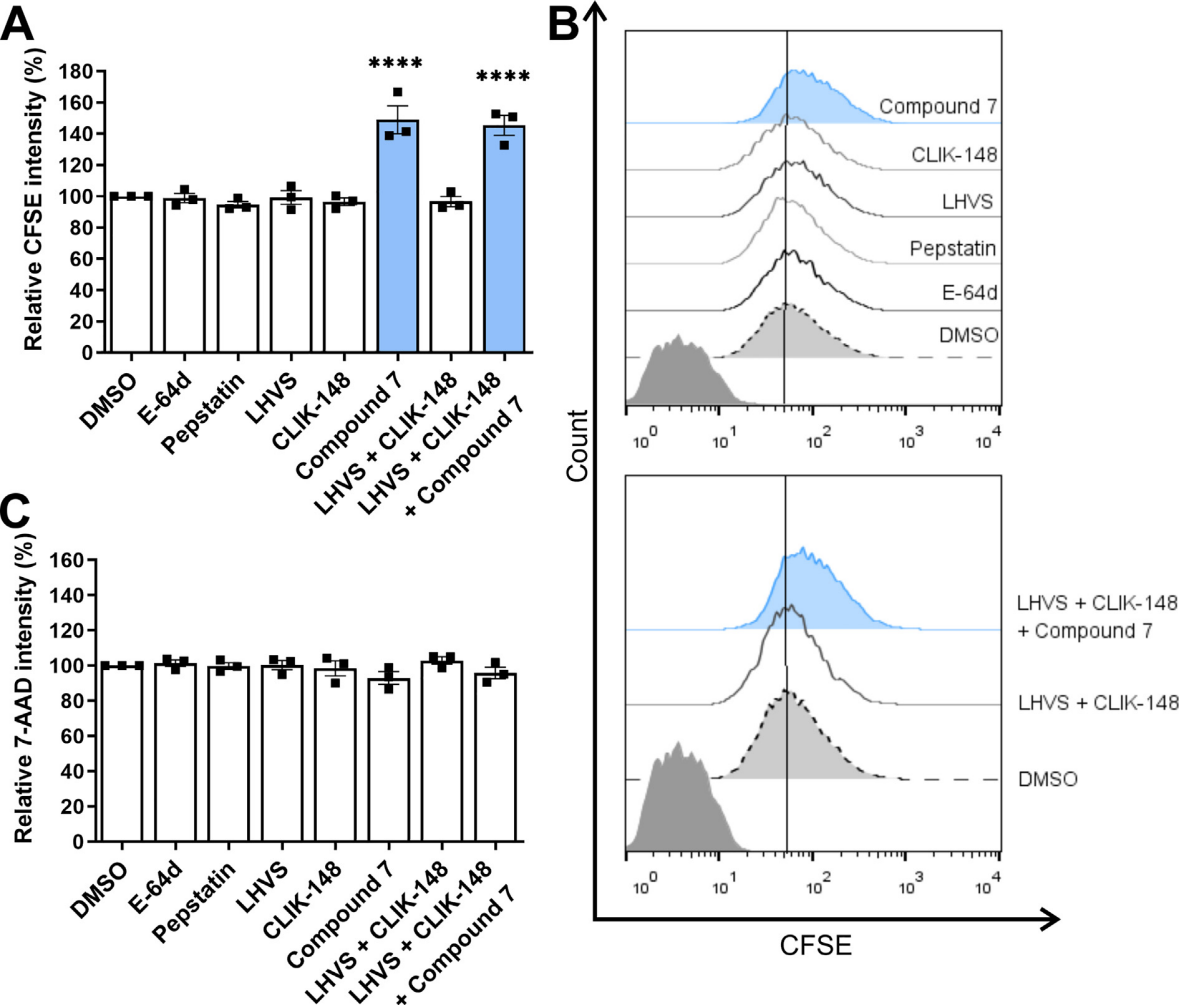
The effect of cathepsin V inhibitors on MCF7 cell proliferation. (A) Cell proliferation monitored as relative carboxyfluorescein succinimidyl ester (CFSE) intensity of CFSE-labeled MCF7 cells in the presence of cathepsin inhibitors compared to DMSO, calculated from data obtained from flow cytometry. (B) CFSE fluorescence intensity after treatment with DMSO (0.1 %, dotted black line, light gray) or E-64d (10 μM), pepstatin (20 μM), LHVS (10 nM), CLIK-148 (1 μM, all solid gray lines), compound **7** (10 μM, blue), or their combinations, as monitored by flow cytometry. Dark gray histograms denote unlabeled cells. (C) The effects of treatments on cell viability as monitored by 7-aminoactinomycin D (7-AAD) staining. The intensity of green CFSE fluorescence was monitored for viable cells only. Data are presented as mean ± SEM (n = 3). ****P < 0.0001 (one-way ANOVA). (For interpretation of the references to colour in this figure legend, the reader is referred to the web version of this article.)

We further evaluated the effects of the most potent selective reversible cathepsin V inhibitor, compound **7**, on tumor cell proliferation using CFSE-labeled MCF7 cells. CFSE stably stains intracellular molecules and is evenly distributed after cell division between the two daughter cells, which thus exhibit half the fluorescence of the parent cells [58]. Therefore, in this assay, lower CFSE signals correlate with higher cell proliferation. The cell-permeant general cysteine cathepsin inhibitor E-64d (10 μM), cathepsin D inhibitor pepstatin (20 μM), cathepsin S inhibitor LHVS (10 nM), and cathepsin L inhibitor CLIK-148 (1 μM), exerted no effects on cell proliferation, as no significant changes in relative CFSE fluorescence intensities were observed compared with DMSO-treated control cells (Fig. 4A and B). However, treatment with compound **7** increased the fluorescence intensity of CFSE-labeled cells by 50.6 ± 5.1 %, compared to control cells (Fig. 4A). A similar effect was observed when combinations of inhibitors were used. Co-treatment with CLIK-148 and LHVS did not change the relative intensity of CFSE-labeled compared to control cells and thus did not affect cell proliferation. Conversely, the addition of compound **7** to the combination (co-treatment with CLIK-148, LHVS, and compound **7** together) increased the relative CFSE fluorescence intensity by 46.1 ± 5.2 %, indicating less cell proliferation (Fig. 4A). This suggests that the effect of compound **7** on cell proliferation is due to the inhibition of cathepsin V and not other related cathepsins. To confirm selective action of the inhibitor on cell proliferation and to exclude non-specific cell toxicity, the cells were additionally stained with 7-AAD before flow cytometry. None of the compounds significantly reduced cell viability at the concentrations tested (Fig. 4C).

### 3.6 Inhibitors impair the elastolytic activity of cathepsin V

**Fig. 5:**
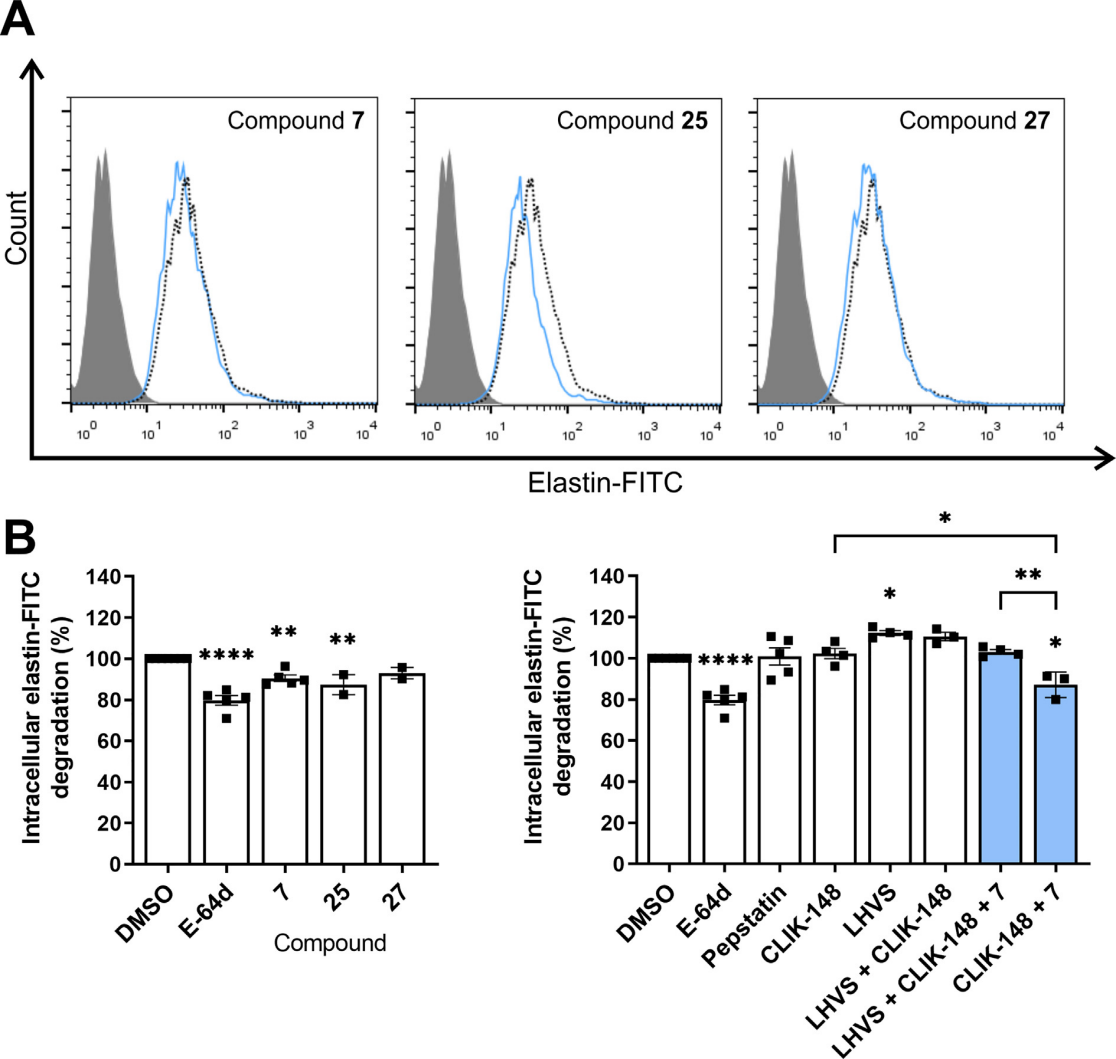
The effect of cathepsin V inhibitors on intracellular elastin-FITC degradation in U-937-derived macrophages. (A) Intracellular elastin-FITC degradation in U-937-derived macrophages (1 × 10^5^) after treatment with DMSO (0.1 %, dotted black line) or compounds **7**, **25**, or **27** (all 10 μM, solid blue line), as monitored by flow cytometry. Gray histograms denote unlabeled cells. (B) Reduction in intracellular elastin-FITC degradation in the presence of E-64d (10 μM), compounds **7**, **25**, or **27** (all 10 μM), pepstatin (20 μM), CLIK-148 (1 μM), LHVS (10 nM), or their combinations, compared with DMSO, as calculated from data obtained by flow cytometry. Data are presented as means ± SEM of at least two independent experiments. *P < 0.05, **P < 0.01, ****P < 0.0001 (one-way ANOVA). (For interpretation of the references to colour in this figure legend, the reader is referred to the web version of this article.)

Cathepsin V has been proposed to expresses intracellular elastolytic activity in activated macrophages [8]. Therefore, the effects of cathepsin V inhibitors on the elastolytic activity of cathepsin V were investigated by monitoring the intracellular degradation of elastin-FITC in macrophage-like cells obtained by PMA differentiation of U-937 cells. Elastin was fluorescently labeled with FITC that, upon proteolytic cleavage, emits a bright green fluorescence, which was quantified by flow cytometry (Fig. 5A). Compounds **7**, **25**, and **27** (10 μM) decreased elastin-FITC degradation by 9.5 ± 1.6 %, 12.6 ± 4.8 %, and 7.1 ± 2.7 %, respectively (Fig. 5B). These results reveal an important contribution of cathepsin V to overall intracellular elastolytic activity, as the degradation of elastin-FITC was inhibited by cell-permeant general cysteine cathepsin inhibitor E-64d by 18.4 ± 7.0 % (10 μM) (Fig. 5B). The cathepsin L- and S-specific inhibitors CLIK-148 and LHVS did not decrease the degradation of elastin-FITC compared to control cells (Fig. 5B). Moreover, the addition of compound **7** (10 μM) to CLIK-148 significantly decreased elastin-FITC degradation compared with control cells and CLIK-148 alone (Fig. 5B). A similar effect was also observed when compound **7** (10 μM) was added to both CLIK-148 and LHVS inhibitors (Fig. 5B).

### 3.7 Cathepsin V inhibition decreases cystatin F activation and increases cytotoxicity of NK-92 and TALL-104 cells

**Fig. 6:**
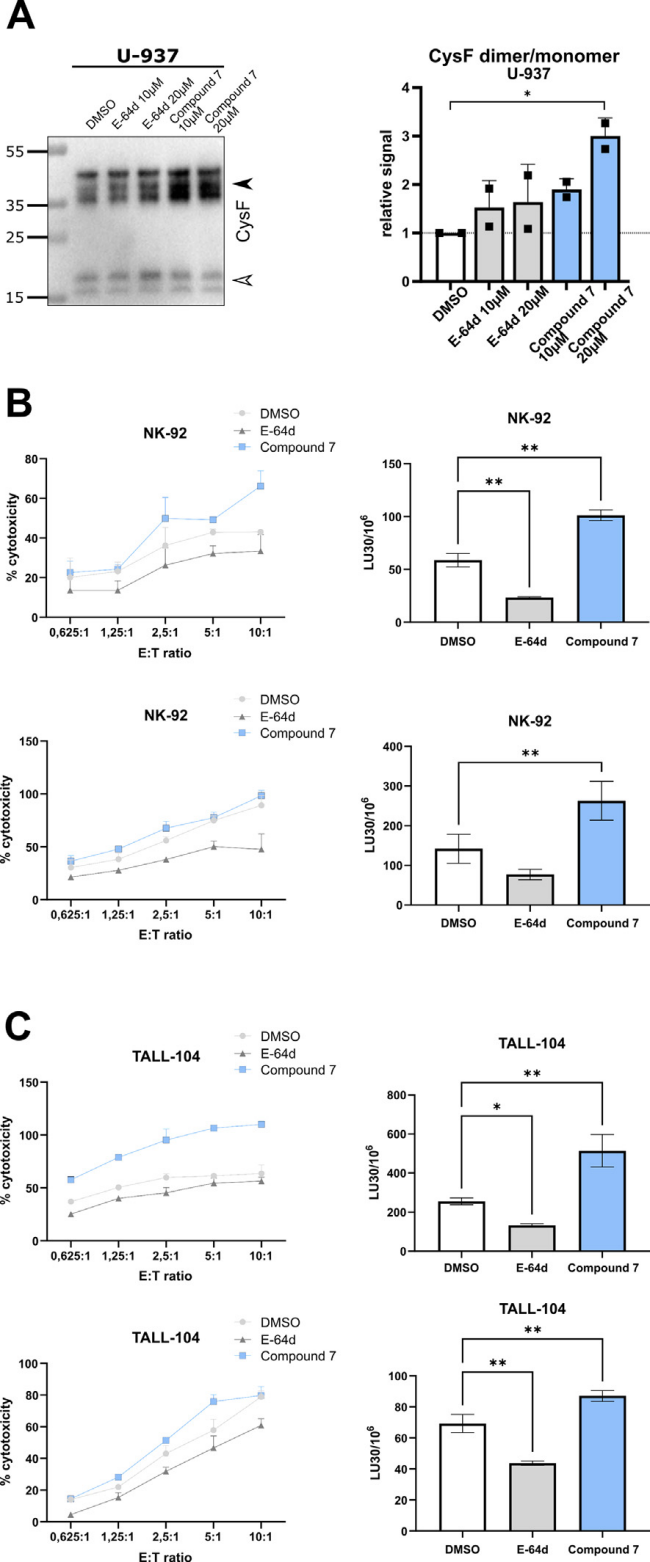
Inhibition of cathepsin V activity decreases cystatin F activation and increases NK-92 and TALL-104 cell cytotoxicity. (A) Representative western blot showing decreased expression of the active cystatin F form in U-937 cells (5 × 10^5^/mL) after treatment with the broad-spectrum peptidase inhibitor E-64d (10 and 20 μM) or the cathepsin V inhibitor compound 7 (10 and 20 μM), compared to DMSO (0.1 %) used as a control. Arrowheads indicate the dimeric (black) and monomeric (white) forms of cystatin F. Multiple bands are due to different *N*-glycosylation forms of cystatin F (left). Relative abundance of the cystatin F dimer/monomer ratio in U-937 cells, normalized to DMSO (0.1 %). Data are presented as means ± SD of at least two independent experiments. (B and C) The cytotoxicity of effector cells NK-92 (B) and TALL-104 (C) on target K-562 cells is decreased after treatment with E-64d (10 μM) but increased after treatment with the specific cathepsin V inhibitor compound **7** (10 μM). Left panels indicate % cytotoxicity determined at different E:T ratios. Right panels indicate lytic units calculated at 30 % cytotoxicity. Data are presented as means ± SD (n = 3). *P < 0.05, **P < 0.01 (one-way ANOVA).

Cathepsin V is involved in processing cystatin F from its inactive dimer to its active monomer by proteolytic cleavage of the 15-amino-acid *N*-terminal peptide. Therefore, we first investigated the effect of cathepsin-V-specific inhibitors on the conversion of dimeric cystatin F to monomeric cystatin F in U-937 cells, which express high cathepsin V levels (Fig. S2). For this purpose, cystatin F dimer-to-monomer ratio was used as it better reflects the changes of cystatin F compared to its monomeric form alone. In cell lysates, the cystatin F dimer-to-monomer ratio was increased after 6 h of treatment with the broad-spectrum peptidase inhibitor E-64d and even further increased after treatment with the selective reversible cathepsin V inhibitor compound **7**, compared to control cell lysates (Fig. 6A). Loading control was performed using StainFree technology (Fig. S3). Multiple bands depicting cystatin F are due to the multiple *N*-glycosylation forms of cystatin F [59].

Cystatin F exerts important regulatory functions in cytotoxic immune cells, and thus we examined the effects of cathepsin V inhibition on cytotoxicity in NK-92 and TALL-104 cells. To exclude the effect of compound **7** on cell viability, we first examined its effect on both cell lines using the MTS cell viability assay. The viability of NK-92 and TALL-104 cells was not significantly reduced after treatment with compound **7** at concentrations up to 10 μM for 24 h (Fig. S4). Similarly, the viability of K-562 cells was not decreased in the presence of compound **7** at concentrations up to 5 μM, whereas cell viability was slightly decreased at 10 μM. Based on these results compound **7** was used in further experiments at a concentration of 10 µM.

As expected, treatment of effector cells (NK-92 or TALL-104) with the broad-spectrum peptidase inhibitor E-64d decreased cytotoxic function, as this inhibitor impairs the activities of cathepsins C, H, and L, which are involved in the activation of granzymes and perforin. However, treatment of NK-92 and TALL-104 cells with compound **7** significantly increased the efficacy of target cell killing by both NK-92 (Fig. 6B) and TALL-104 cells (Fig. 6C). The selective inhibition of cathepsin V prevents the monomerization and activation of cystatin F, decreasing the inhibitory effect of cystatin F on the cytotoxic activity of effector cells.

## 4 Discussion

Cathepsin V has been proposed as a promising therapeutic target because of its restricted expression under physiological conditions and specific functions during pathological processes in cancerous, cardiovascular, and neurological diseases [3], [4], [23], [24]. To validate its therapeutic potential, new potent, reversible, and, importantly, selective (due to its high similarity with other cathepsins) inhibitors are required. In this study, we used virtual high-throughput screening of commercially available compound libraries followed by biological evaluation to identify compounds that could be used as leads for further development of cathepsin-V-selective inhibitors. The best hits were further evaluated in cell-based *in vitro* functional assays that assessed whether the hits impaired cathepsin-V-dependent pathological processes.

Potential cathepsin V inhibitors were screened via two parallel docking approaches: noncovalent classic molecular docking and covalent molecular docking using compounds with epoxide warheads. The compounds with the best scores were selected for further biological evaluation. Using enzyme kinetic methods, we determined their relative inhibition, i.e., the inhibition constants and inhibition modes against recombinant cathepsin V. Considering the large similarities between cathepsins V and L [3], [4], [5], we also tested the compounds for cathepsin L inhibition. Inhibitor selectivity against these two enzymes is an important issue to distinguish between specific roles during physiological and pathological processes as well as to avoid off-target effects during potential therapeutic application [60].

**Fig. 7:**
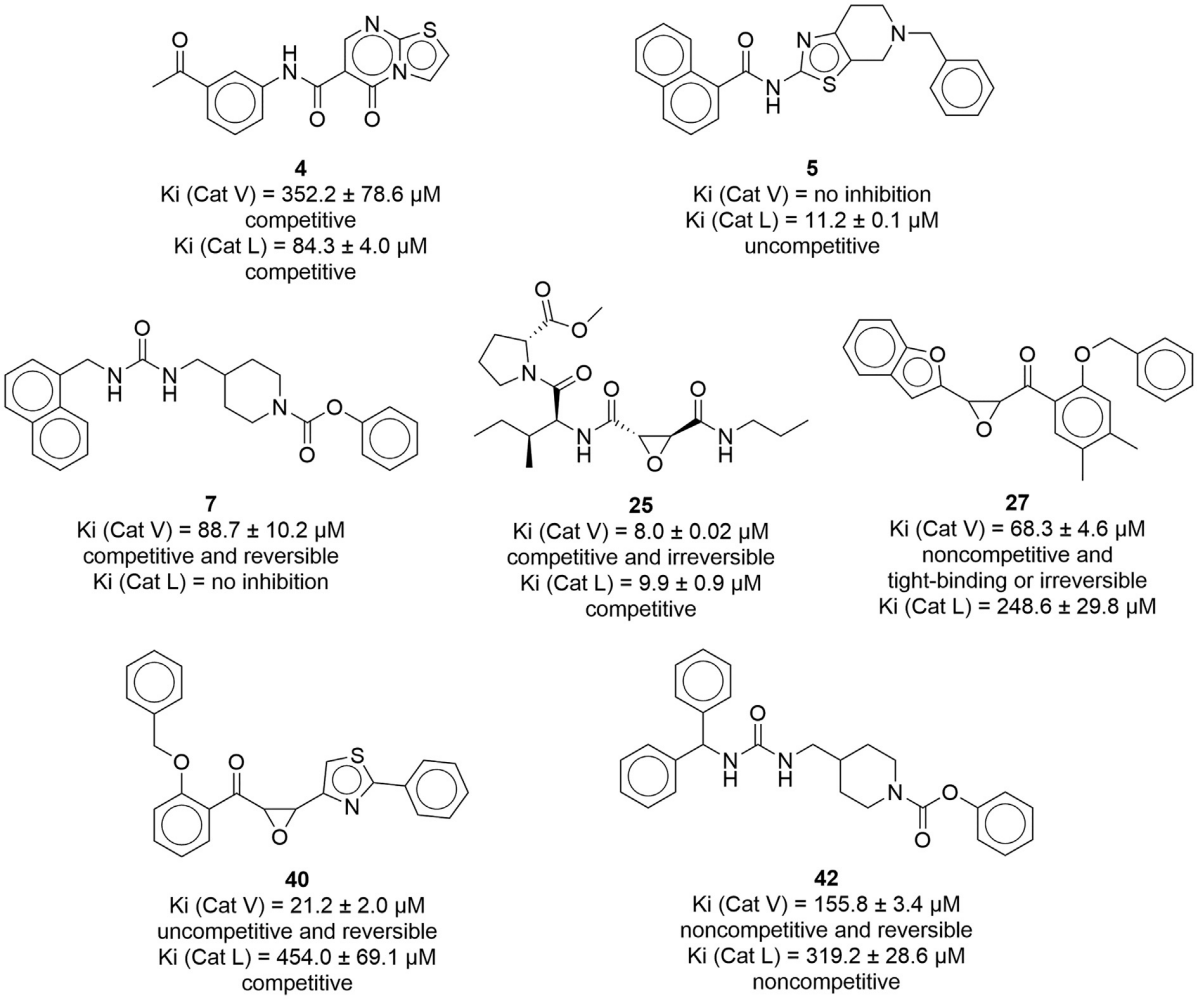
The best-performing inhibitors identified during this study.

The best cathepsin V inhibitors with the lowest constants of inhibition among the tested compounds were compounds **25**, **27**, and **7**. Conversely, compounds **25**, **5**, and **4** were the most potent inhibitors of cathepsin L (Fig. 7). The most potent cathepsin V inhibitor, compound **25**, was also the most potent cathepsin L inhibitor, therefore lacking the demanded selectivity. This dual action can be explained by the tripeptide-mimic structural nature of compound **25** that incorporates an epoxide warhead between the Pro-Ile and propylamine motifs. Compound **25** occupies the S1′ pocket of cathepsin V with the terminal propyl S2 pocket with the Ile mimic, while positioning its methoxycarbonyl substructure on the other distal side of the molecule, above the S3 pocket, where it does not maintain any key contacts. Namely, pockets S1 and S3 of cathepsin V are nonspecific (cathepsin L shows preference for positively charged residues), whereas the S2 pocket of both cathepsins V and L has a selectivity towards hydrophobic residues with a preference for Phe and Leu. As the S1′ pocket of both cathepsins V and L is nonspecific, compound **25** hardly exerts selectivity via its structural features. Moreover, data obtained from the dilution assay suggest irreversible binding of compound **25** to cathepsin V, in accordance with its epoxy warhead, similar to E-64, which forms a covalent bond with the thiol group of the active site of cysteine [61].

Although cathepsins L and V have very similar structures, they have markedly different surface electrostatic potentials [4]. Whereas the electrostatic potential of cathepsin L is negative over extended regions of the surface, including the active site, cathepsin V has only a few negative areas, some positive areas, and approximately neutral areas around the active site cleft. This structural feature is utilized by the compound **27**. Compound **27** differs from compound **25** in its structure. It also incorporates an epoxide warhead; however, the latter is attached to a lipophilic benzofuran system on one side and a [2-(benzyloxy)-4,5-xylyl]methanone system on the other terminal side. Three bulky aromatic systems could explain its increased selectivity for cathepsin V compared to cathepsin L. Compound **27** fully occupies the S2 pocket and covers the central gorge with its lipophilic [2-(benzyloxy)-4,5-xylyl]methanone system, confirming that the cathepsin V tends to form primary hydrophobic interactions. Conversely, also electrostatic components are indicated for cathepsin L [4]. A similar behavior and binding mode were also exerted by compound **26**, which replaces the central xylene linker with a similar lipophilic bromophenyl moiety. Due to the presence of an electrophilic epoxide in the structure, only partial cathepsin V recovery after dilution following 2 h incubation with compound **27** could be achieved, suggesting that compound **27** is a covalent inhibitor.

Compound **7** also showed high selectivity for cathepsin V over cathepsin L. Compound **7** is a urea derivative in which the moiety serves as a central linker. Its noncovalent nature was confirmed by a dilution assay, in which the activity of cathepsin V was recovered after 2 h of incubation followed by rapid dilution. Due to its structural similarity and analogous binding mode to compound **27**, compound **7** may similarly occupy the S2 binding pocket of cathepsin V with its naphthyl moiety (benzofuran in compound **27**) and analogously reach the S1′ binding pocket with its phenylformylate structural element (benzyloxy in compound **27**). A similar steric configuration of these aromatic systems could confer its selectivity, while its central ureidomethyl-piperidine anchors it near the catalytic Cys25 of cathepsin V via an H-bond to Asp162.

Similarly, to compound **7**, compounds **4** and **5** (which inhibited cathepsin L but not cathepsin V) do not have any highly reactive functional groups in their structure and indicated reversible inhibition in the dilution assay. Structurally, compounds **4** and **5** are carboxamides and differ from compounds **7** and **27**, which were selective for cathepsin V. Taken together, these data also demonstrate obvious differences in selectivity between noncovalent reversible inhibitors (compound **7** for cathepsin V and compounds **4** and **5** selective for cathepsin L) compared to covalent irreversible inhibitors (compounds **25** and **27**). Reversible inhibitors are particularly desirable in the development of new inhibitors as lead compounds that could be used in clinical practice because they avoid side effects that might occur due to the high reactivity and covalent bond formation of irreversible inhibitors [60]. However, potent, selective, covalent, irreversible inhibitors remain attractive molecular tools for investigating the role of cathepsin V in pathological processes in cells.

To further improve the inhibition of cathepsin V and to identify structural elements important for cathepsin V inhibition and selectivity, further ligand-based screening of commercially available compound libraries was performed based on the structural properties of compounds with the lowest inhibition constant for cathepsin V. The most potent inhibitors were compounds **40** and **42**. Structurally similar to compound **27** from the previous group, compound **40** has a reactive epoxide ring but also a triazole heterocycle coupled to the benzene ring, whereas the phenyl ring, which is attached to the epoxide ring via a carboxyl group, does not contain methyl groups. Interestingly, full recovery of cathepsin V activity was observed for compound **40** in the dilution assay, suggesting that compound **40** acts as a reversible inhibitor despite its reactive epoxide group. These structural modifications appear to increase the potency and selectivity for cathepsin V compared to cathepsin L. Conversely, similar to compound **7**, compound **42** is a urea derivative without a highly reactive structural element and reversibly inhibits cathepsin V. Replacement of the naphthyl group in compound **7** with two phenyl groups in compound **42** decreased the selectivity and potency of cathepsin V inhibition. Moreover, the lower activities of compound **40** (in which the naphthyl group was replaced by fluorobenzene) and compound **47** (in which the naphthyl group was replaced by methyl phenyl ether), indicate that a larger planar group attached to the urea fragment is beneficial for selective inhibition of cathepsin V. Furthermore, compound **44** exhibited lower activity than compound **7**, suggesting that a larger fragment on the piperidine ring, such as the benzene ring in compound **7**, is preferred for cathepsin V inhibition. This is in agreement with previous observations on cathepsin V electrostatic potential and substrate specificity [4], [5].

Next, we evaluated the strongest cathepsin V inhibitors for their ability to impair cathepsin V activity in cell models that mimic the pathological processes in which cathepsin V is involved. Several studies have suggested that cathepsin V contributes to tumor cell hyperproliferation [3], [18], [20], [24]. In view of this, we evaluated the effect of cathepsin V inhibition on tumor cell proliferation. Human epithelial mammary gland adenocarcinoma cells MCF7 were used to monitor cell proliferation because silencing cathepsin V inhibits their growth [62]. Cathepsin V is closely related to cathepsin L [2], [3], [4], and the substrate specificity of the substrate binding pocket S2 of cathepsin V is intermediate between those of cathepsins S and L [4]. Thus, cathepsin L and S and general cathepsin inhibitors were included as controls in this study to rule out off-target effects on closely related enzymes and to confirm that the inhibitory effects were truly due to cathepsin V. The cathepsin V inhibitor compound **7** significantly decreased tumor cell proliferation. By contrast, the cell-permeant general cysteine cathepsin inhibitor E-64d, cathepsin L inhibitor CLIK-148, and cathepsin S inhibitor LHVS did not decrease tumor cell proliferation. In addition, cell proliferation was inhibited when cells were treated with the combination of compound **7**, CLIK-148, and LHVS, but not with the combination of CLIK-148 and LHVS. This confirms that cathepsin V plays a role in tumor cell proliferation, whereas cathepsins L and S do not.

Cathepsin V has been shown to exhibit the most potent elastase activity among human cathepsins [8]. Previously, by using inhibitors that distinguish between the elastolytic activity of cathepsins, namely cathepsins K and S and cathepsin L-like cysteine peptidases, cathepsin V was shown to contribute up to 20 % of the total cysteine peptidase-dependent elastolytic activity in macrophages [8]. Cathepsin V is particularly important for intracellular elastin degradation in macrophages, providing one-third of the total 60 % intracellular elastolytic activity attributed to cysteine cathepsins [8]. As a macrophage model, we used U-937 cells, differentiated by PMA, and monitored intracellular degradation of FITC-labeled elastin that was internalized into the cells. Compounds **7**, **25**, and **27** all decreased elastin degradation in U-937 macrophage-like cells. Again, to exclude the contribution of other cathepsins with elastase activity, the effect of compound **7** was compared to CLIK-148 and LHVS, selective inhibitors of cathepsins L and S, respectively. Compound **7** inhibited elastin degradation significantly more than CLIK-148 or LHVS. The effects of different combinations of these inhibitors were evaluated as well. Elastin degradation was decreased when compound **7** was added to CLIK-148 or to the combination of CLIK-148 and LHVS. Our results demonstrate that besides the general cathepsin inhibitor E-64d, only cathepsin V inhibitors significantly decreased intracellular elastin degradation, whereas cathepsin L and S inhibitors do not affect it. Together, these results confirm an important contribution of cathepsin V to intracellular elastin degradation.

Another important function of cathepsin V is its regulation of the cytotoxicity of immune cells, such as NK cells and CTLs. In these cells, cystatin F, an endogenous inhibitor of cysteine cathepsins and a member of the type II cystatin family, has been recognized as an important mediator of immunosuppression [28], [30], [63], [64]. Cystatin F in these cells regulates the activities of cathepsins C, H, and L, the main enzymes that convert granzymes and perforin from their precursor forms [28], [29], [33]. Cystatin F is synthesized as an inactive dimer, and its monomerization within the *endo*-lysosomal pathway is a prerequisite for its activity [65]. Proteolytic cleavage of the 15-amino-acid *N*-terminal peptide by cathepsin V [11] enhances cystatin F monomerization, promoting the inhibition of cathepsins C, H, and L and consequently impairing cell cytotoxicity [28], [29], [33]. Selective cathepsin V inhibitors could thus reverse the immunosuppressive function of cystatin F, not affecting the role of other related cathepsins involved in cytotoxic function. Therefore, we evaluated whether the selective cathepsin V inhibitor, compound **7**, can impair cystatin F processing and further examined the effect of cathepsin V inhibition on the cytotoxic activity of NK cells and CTLs. In U-937 cells, we showed that cathepsin V inhibition by compound **7** decreased the conversion of the inactive dimeric cystatin F to its active monomeric form. In addition, treatment of NK-92 and TALL-104 effector cytotoxic immune cells with compound **7**, significantly increased their potential to kill target K-562 cells. This effect is in contrast with that of general cathepsin inhibitor E-64d which decreased cytotoxic function, as it impairs the activities of cathepsins C, H, and L, involved in the activation of granzymes and perforin. As NK cells and CTLs are the main effector cells in the antitumor immune response, selective cathepsin V inhibitors are candidates to be included in protocols of cancer immunotherapy.

## 5 Conclusion

Taken together, we have identified and characterized novel, potent, selective inhibitors of cathepsin V that interfere with processes of tumor progression in which cathepsin V is involved. We demonstrated that the ureido methylpiperidine carboxylate derivative, compound **7**, is a reversible, selective, and potent inhibitor of cathepsin V with the most preferable characteristics for further evaluation. *In vitro* functional assays revealed that compound **7** significantly affects cell proliferation and elastin degradation and increases the cytotoxicity of immune cells by impairing the conversion of cystatin F from its inactive dimeric to its active monomeric form. Our results therefore confirm that cathepsin V is a potential target for improving cancer therapy and provide lead compounds for further development and optimization.

## 6 Associated content

Additional file. SMILES Document.

## 7 Authors' contributions

A. Mitrović, M. Jukič, M. Perišić Nanut, S. Gobec and J. Kos conceived and designed the study; A. Mitrović and L. Bolčina performed enzyme kinetic assays; A. Mitrović, E. Senjor, L. Bolčina, and M. Prunk performed cell assays; M. Jukič and M. Proj performed high-throughput virtual screening, molecular docking experiments and compound evaluation experiments; A. Mitrović, E. Senjor, M. Jukič, M. Prunk, M. Proj and M. Perišić Nanut analyzed the data; M. Perišić Nanut, S. Gobec and J. Kos supervised the study; A. Mitrović, E. Senjor, M. Jukič, M. Proj and J. Kos wrote the manuscript. All authors revised (review and edited) the manuscript. All authors read and approved the final manuscript.

## Funding

This work was supported by the Slovenian Research Agency, Grant No J4-1776 and P4-0127 to J.K., J3-3071 to A.M., J3-2516 to M.P.N., and P1-0208 to S.G. The funders had no role in the study design, data collection and analysis, or preparation of the manuscript.

## CRediT authorship contribution statement

**Ana Mitrović:** Conceptualization, Methodology, Validation, Formal analysis, Investigation, Writing – original draft, Visualization, Project administration, Funding acquisition. **Emanuela Senjor:** Methodology, Validation, Formal analysis, Investigation, Writing – original draft, Visualization. **Marko Jukić:** Methodology, Validation, Formal analysis, Investigation, Writing – original draft, Visualization. **Lara Bolčina:** Investigation, Writing – review & editing. **Mateja Prunk:** Methodology, Validation, Formal analysis, Investigation, Writing – review & editing. **Matic Proj:** Validation, Formal analysis, Investigation, Writing – original draft, Visualization. **Milica Perišić Nanut:** Conceptualization, Validation, Formal analysis, Writing – review & editing, Supervision, Funding acquisition. **Stanislav Gobec:** Conceptualization, Writing – review & editing, Supervision, Funding acquisition. **Janko Kos:** Conceptualization, Writing – review & editing, Supervision, Project administration, Funding acquisition.

## Declaration of Competing Interest

The authors declare that they have no known competing financial interests or personal relationships that could have appeared to influence the work reported in this paper.
