# Supplementary material for: New inhibitors of cathepsin V impair tumor cell proliferation and elastin degradation and increase immune cell cytotoxicity
Source: Comput Struct Biotechnol J. 2022 Aug 28;20:4667–87. doi: 10.1016/j.csbj.2022.08.046 (PMC9459403; doi:10.1016/j.csbj.2022.08.046)

# Supporting Information

# New inhibitors of cathepsin V impair tumor cell proliferation and elastin degradation and increase immune cell cytotoxicity

Ana Mitrović^a,b,*^, Emanuela Senjor^a,b^, Marko Jukić^b^, Lara Bolčina^a,b^, Mateja Prunk^a^, Matic Proj^b^, Milica Perišić Nanut^a^, Stanislav Gobec^b^, Janko Kos^a,b^

^a^Department of Biotechnology, Jožef Stefan Institute, Jamova 39, 1000 Ljubljana, Slovenia

^b^Faculty of Pharmacy, University of Ljubljana, Aškerčeva cesta 7, 1000 Ljubljana, Slovenia

^*^Corresponding author:

Ana Mitrović, Department of Biotechnology, Jožef Stefan Institute, Jamova 39, 1000 Ljubljana, Slovenia, [ana.mitrovic@ijs.si](mailto:ana.mitrovic@ijs.si), phone: +386 1 477 3754; ORCHID: 0000-0002-6996-4831

## Supporting Figures

**Figure S1.** (Data from the) Aqueous stability of compound **7** in HPLC-based assay (for compound 7). The analyte AUCs were normalized on internal standard AUCs. The difference between the last and the first timepoint was smaller than the experimental error (approximately 2%).

**
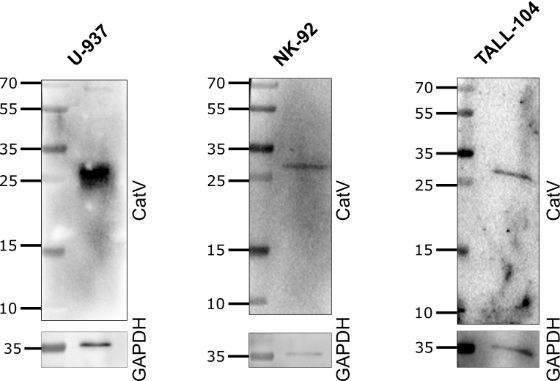
**

**Figure S2.** Cathepsin V protein levels in U-937, NK-92, and TALL-104 cells as determined by western blot analysis.

**
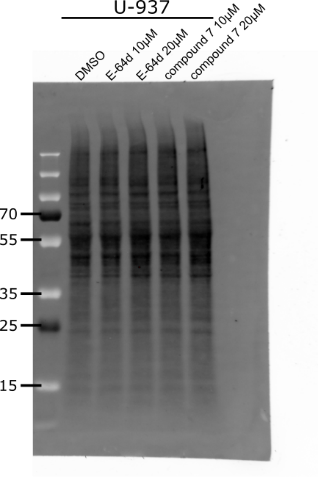
**

**Figure S3.** Stain-free loading control. The western blot shown in Figure 6A was quantified according to the total amount of protein using Stain-Free technology (Biorad).

**
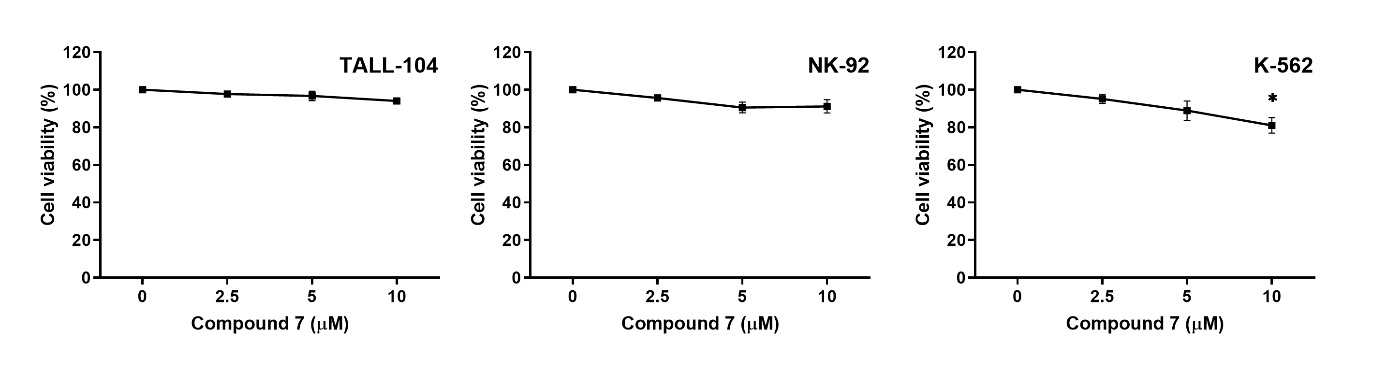
**

**Figure S4.** The effect of compound **7** on cell viability. NK-92 (2.5 × 10^4^), TALL-104 (1 × 10^5^), and K-562 (1 × 10^4^) cells were treated with increasing concentrations of compound **7** for 24 h. Cell viability was determined by the MTS assay. Data are presented as the percentage of viable cells (mean ± SEM) from at least two independent experiments, each performed in quadruplicate. **P* < 0.05 (one-way ANOVA).

## NMR spectra

**4**

**
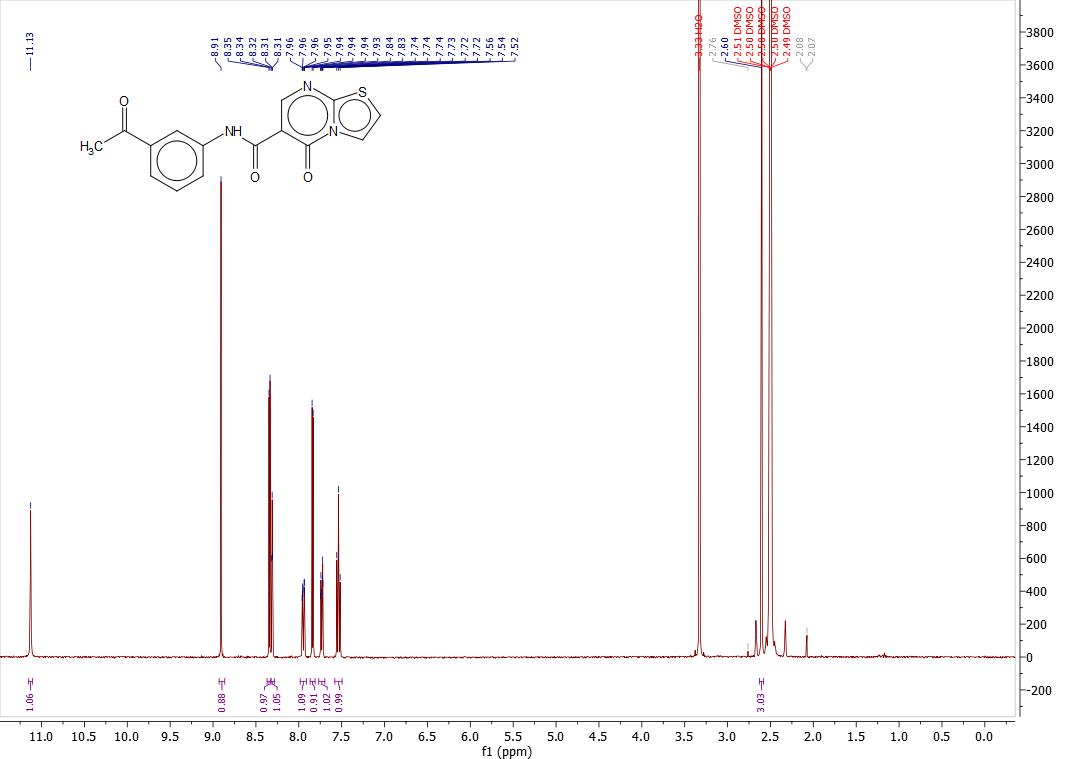
**

**5**

**
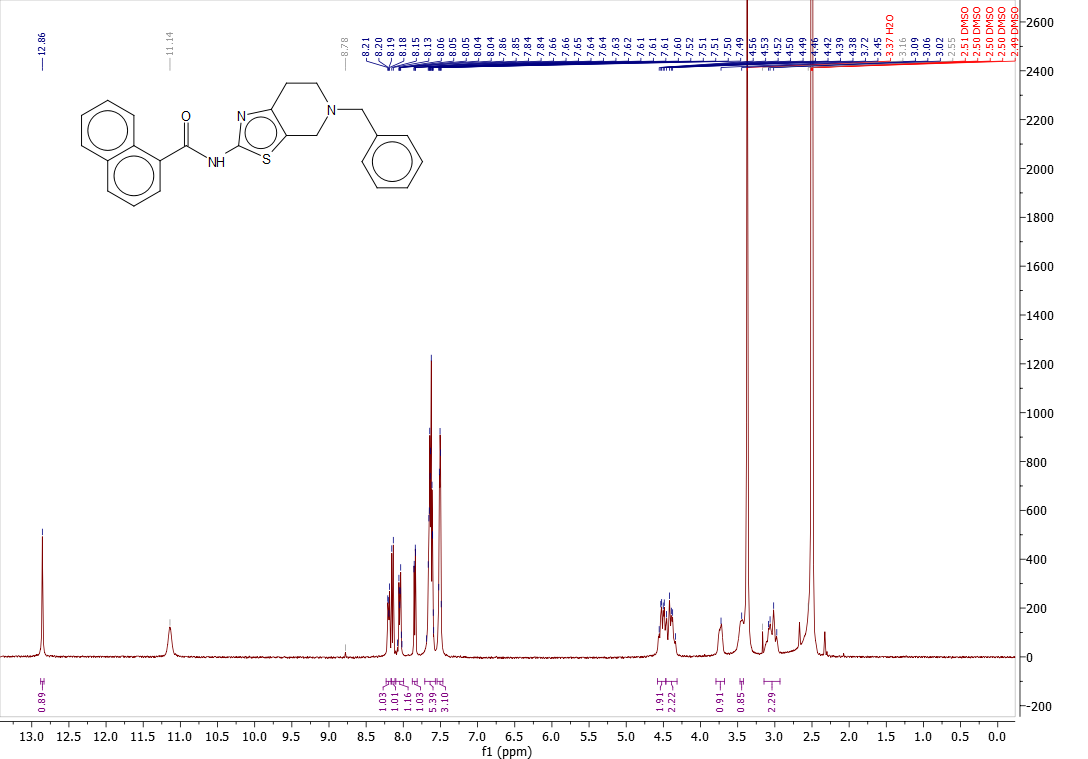
**

**7**


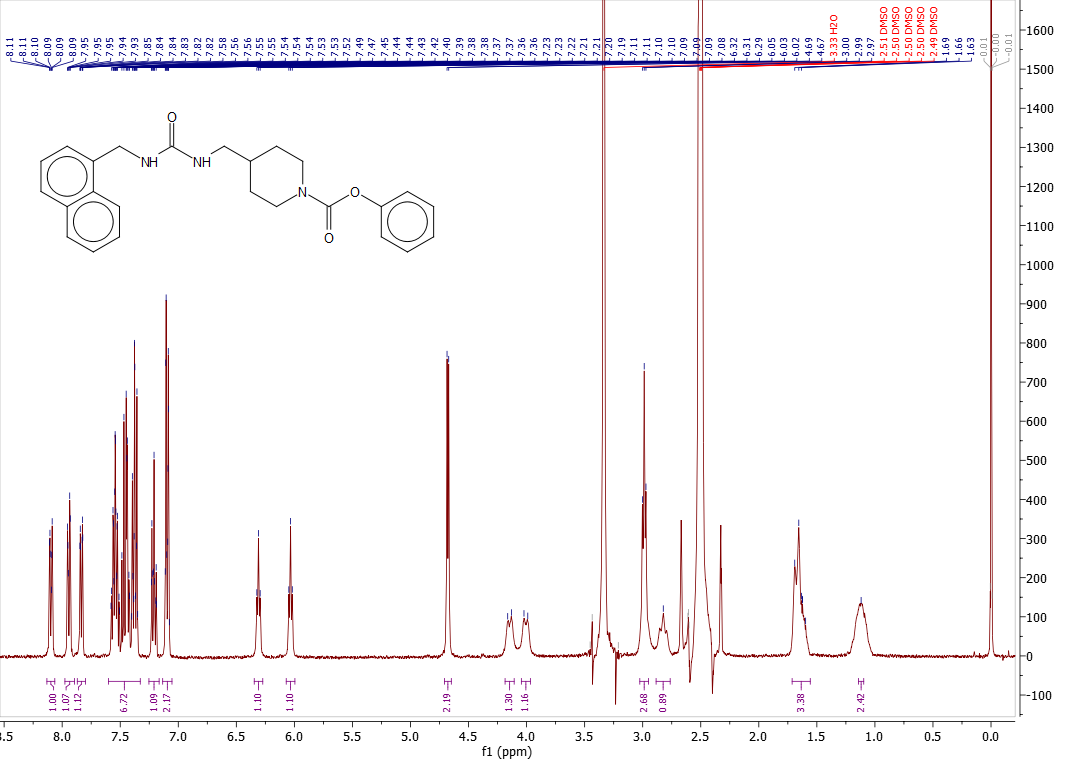


**25**

**
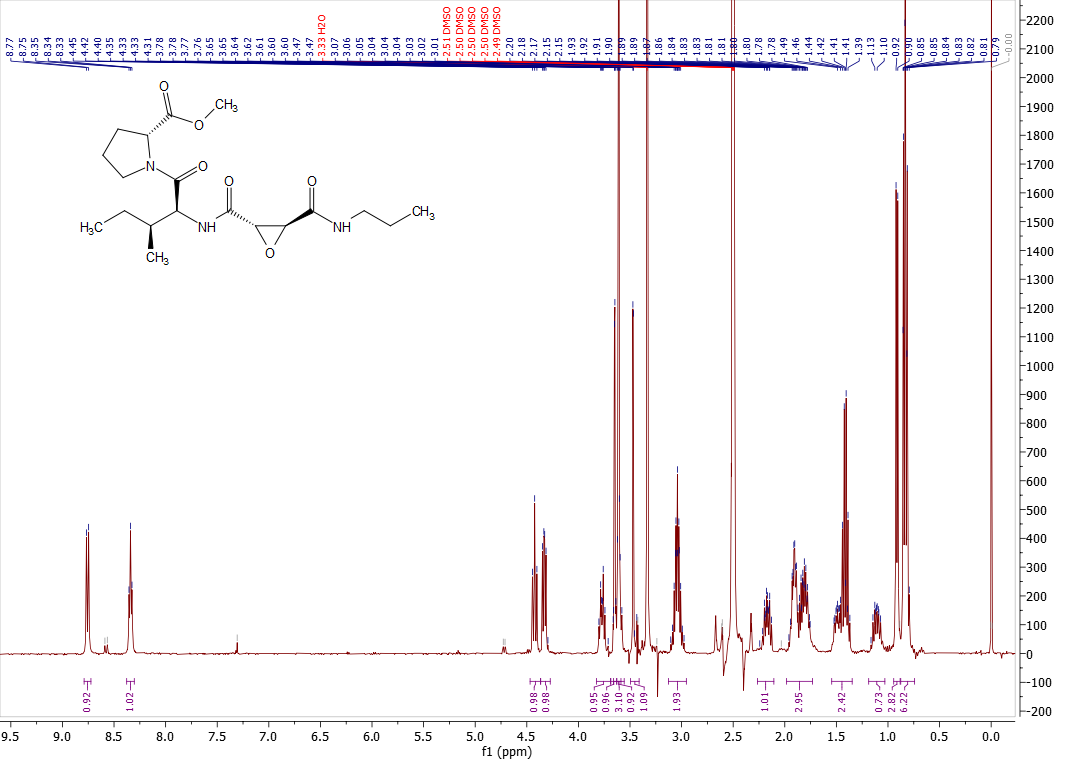
**

**27**


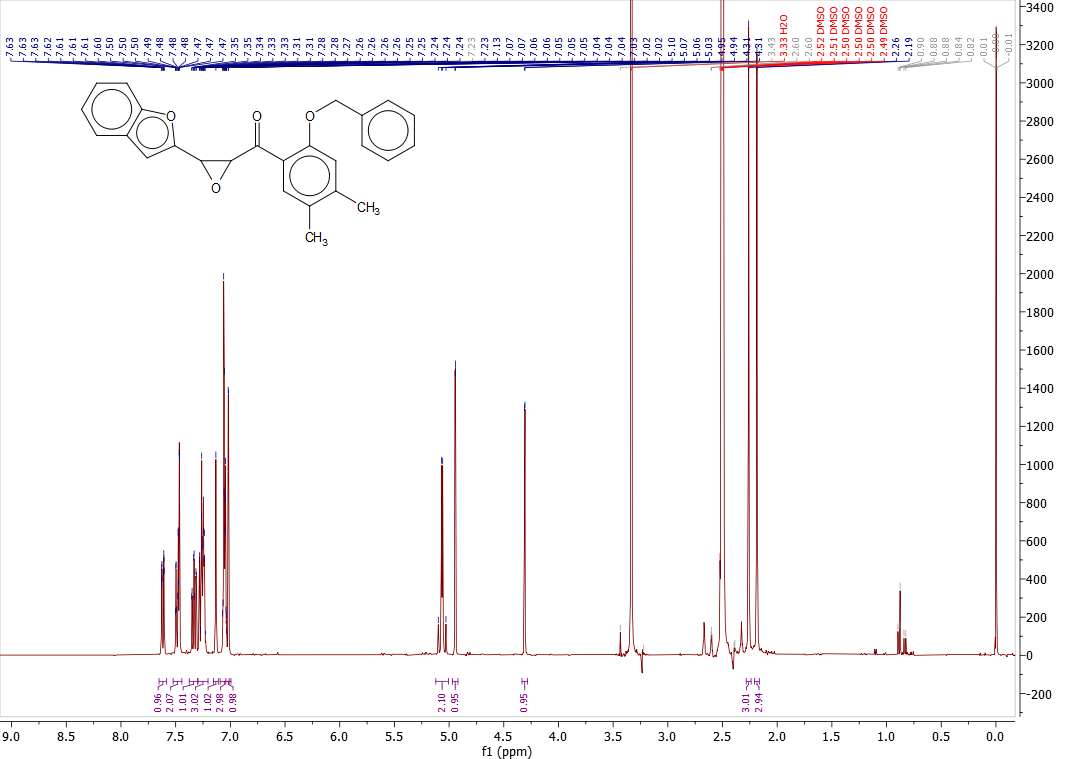


**40**


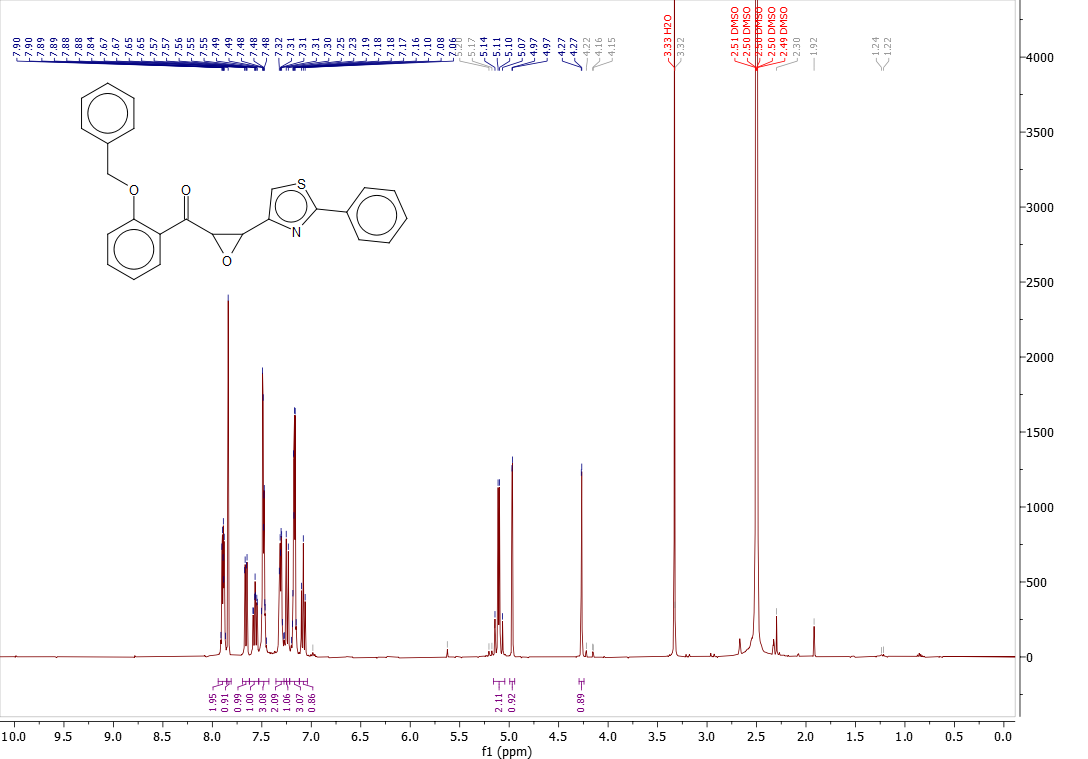


**42**

**
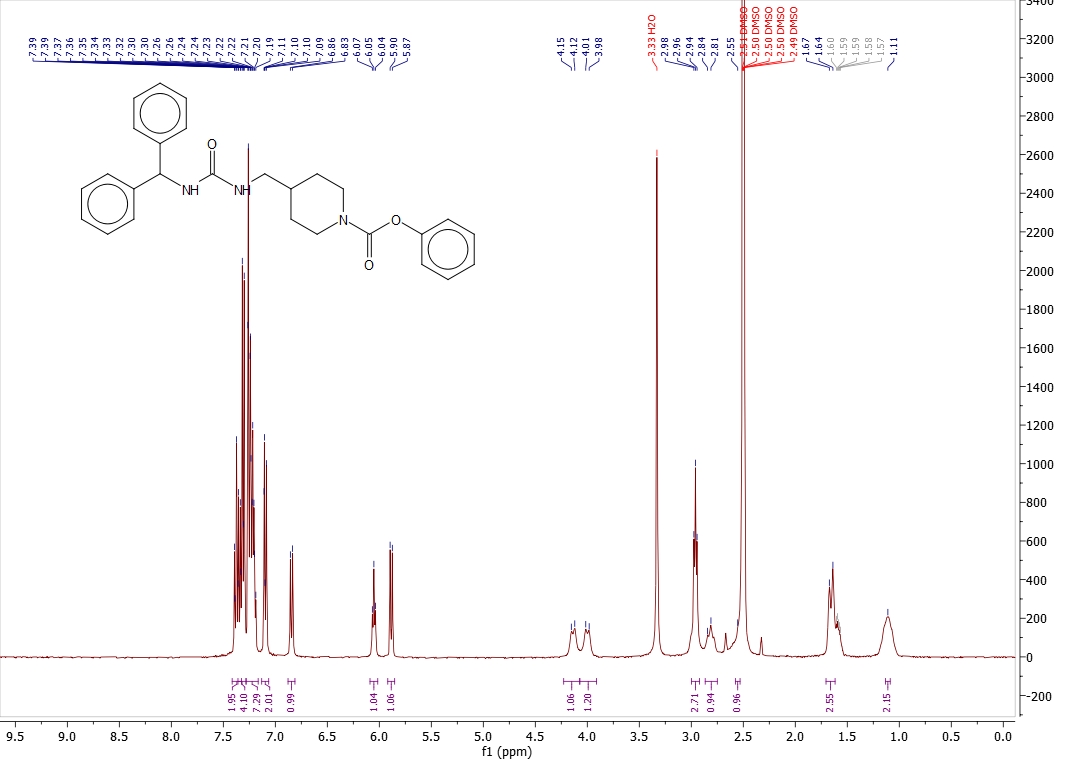
**

## HPLC traces

**4**


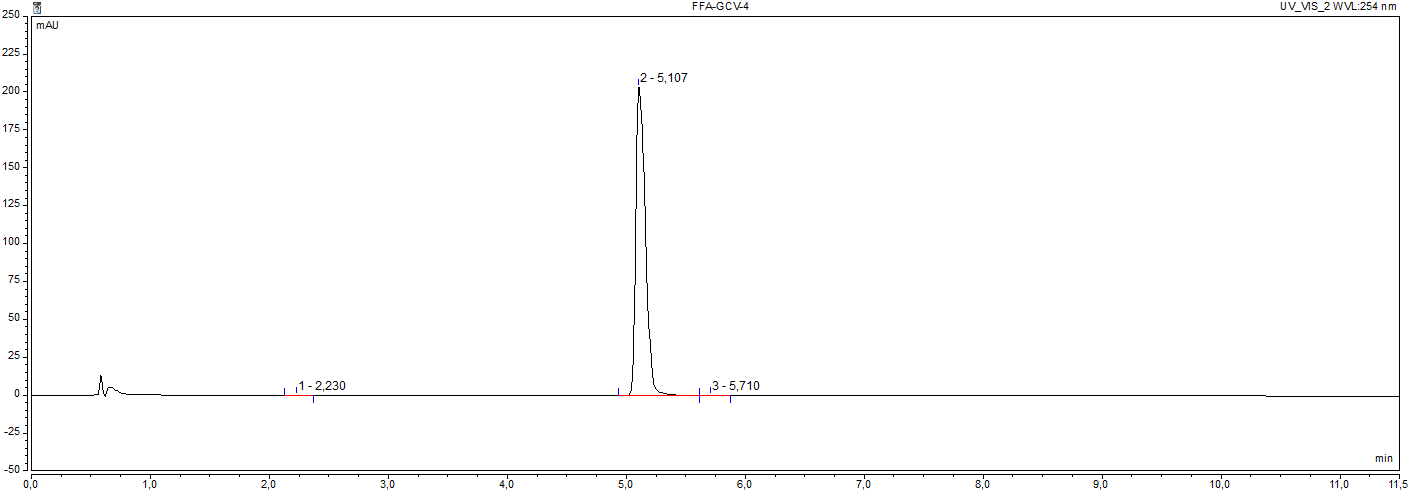


**5**


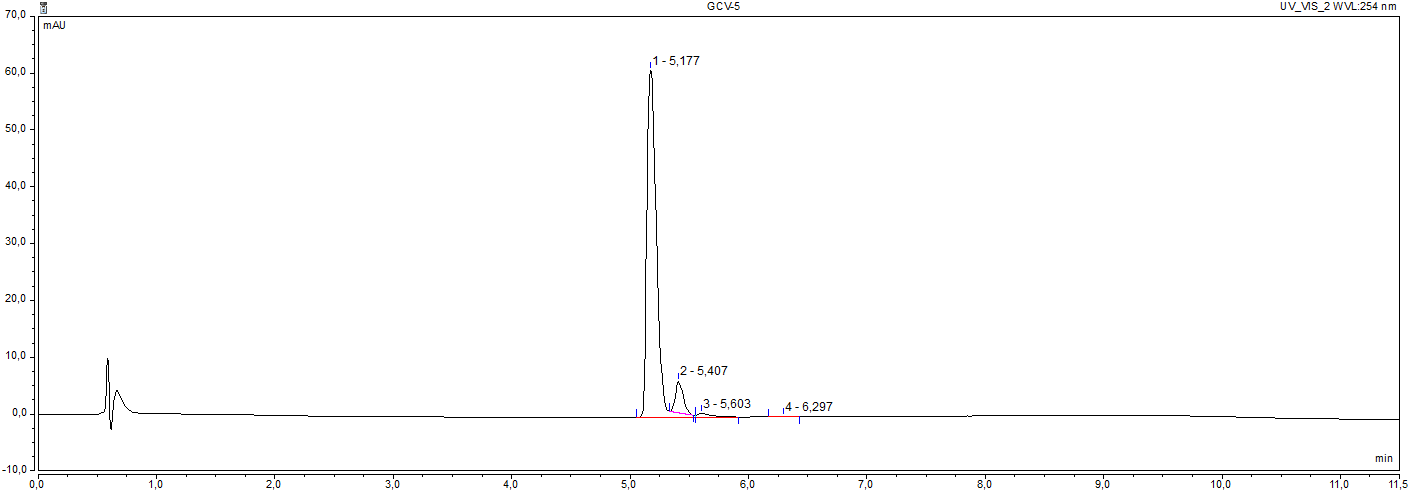


**7**


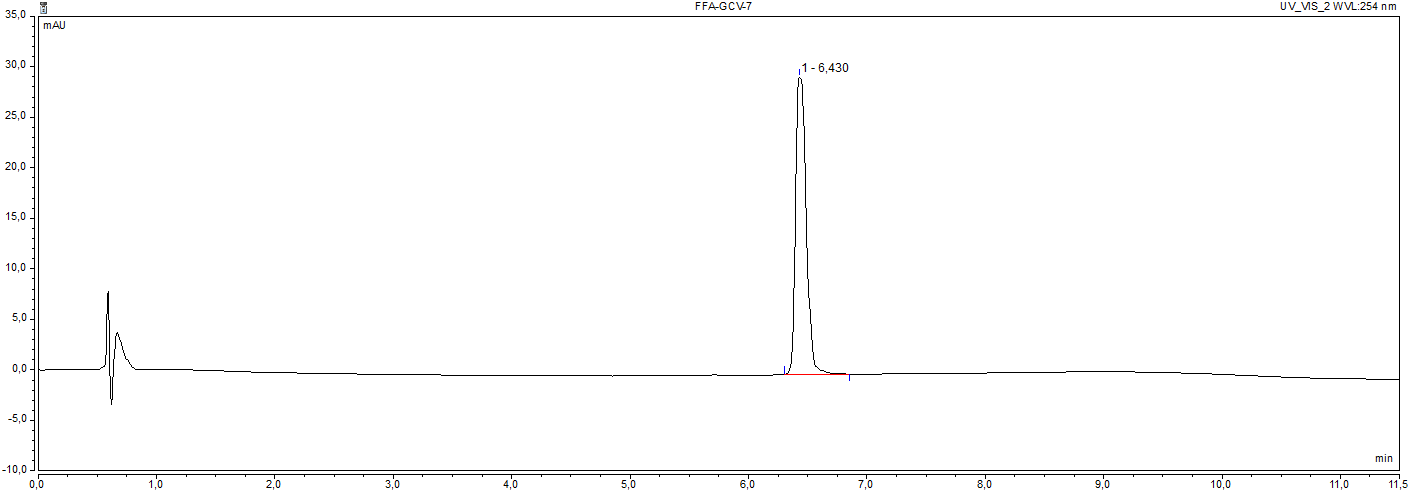


**25**


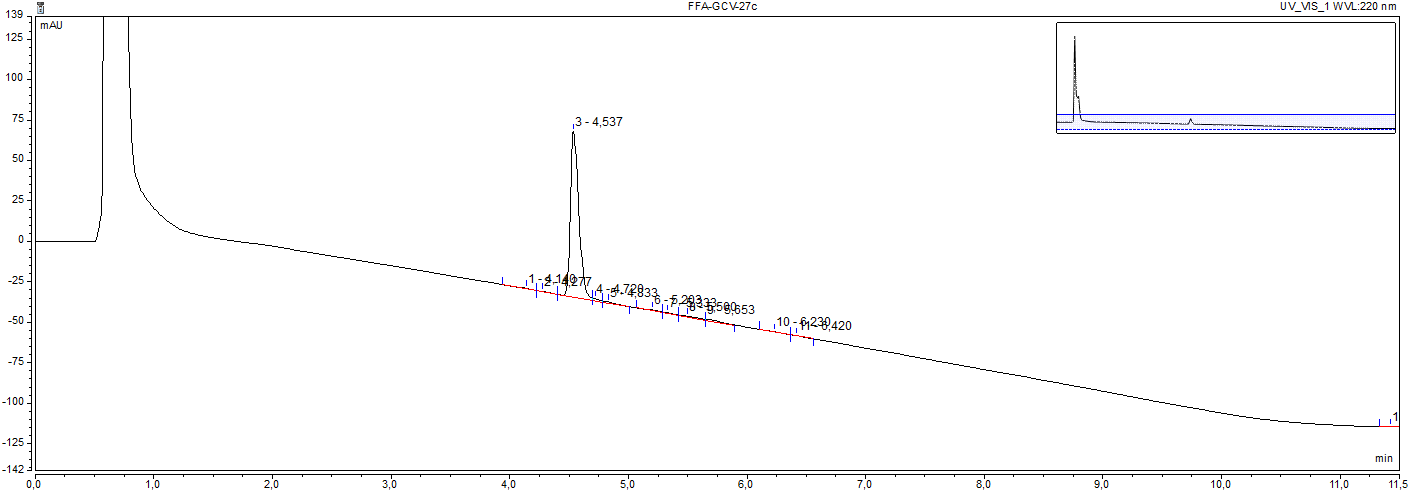


**40**


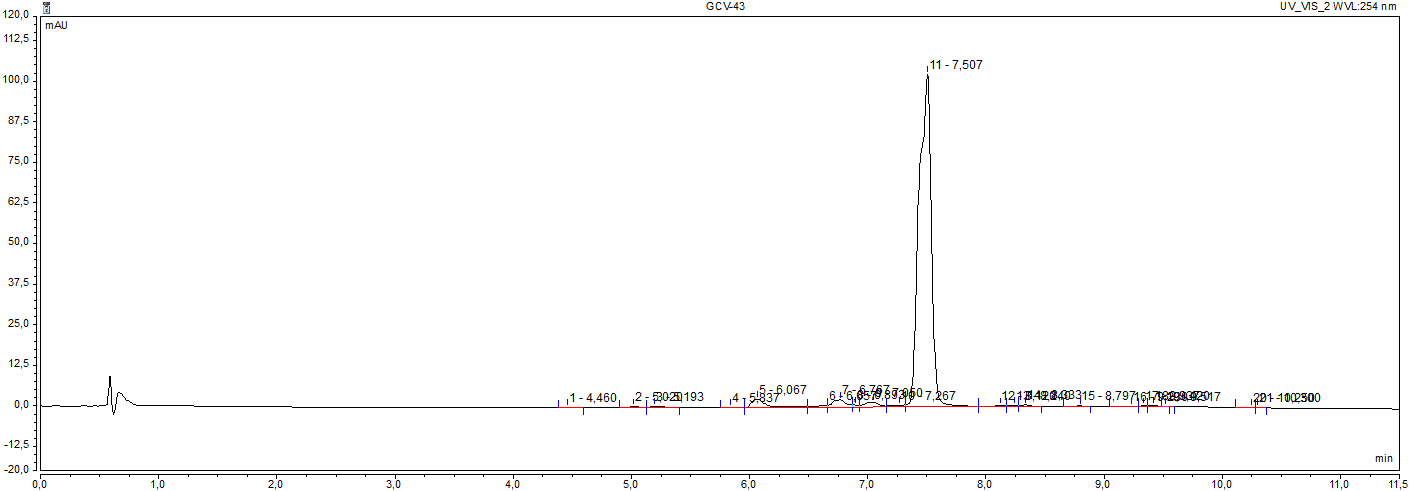


**42**


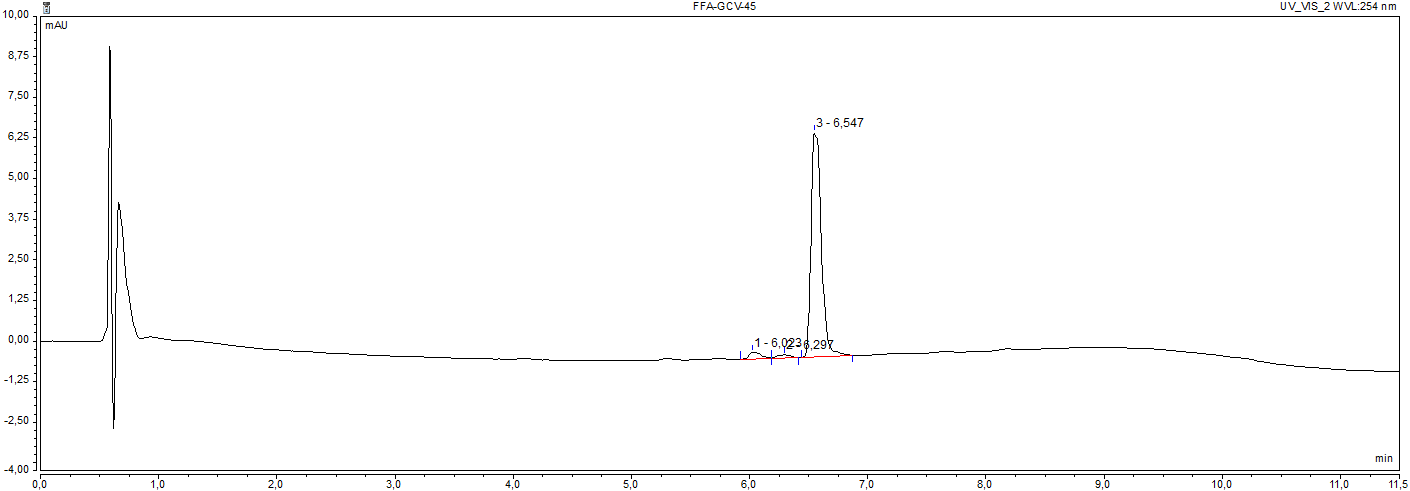

Supplement: Supplementary data 1 [file mmc1.docx]
